# Competing fairness ideals underlie wealth inequality across decision contexts

**DOI:** 10.1038/s41598-024-83361-z

**Published:** 2024-12-30

**Authors:** Inge Huijsmans, Sarah Vahed, Cătălina E. Răţală, Alberto Llera, Alan G. Sanfey

**Affiliations:** 1 Donders Institute for Brain, Cognition and Behavior, Radboud University, Nijmegen, The Netherlands; 2 Department of Cognitive Neuroscience, Radboud University Nijmegen Medical Centre, Nijmegen, Netherlands; 3 Behavioural Science Institute, Radboud University, Nijmegen, The Netherlands

**Keywords:** Wealth inequality, Fairness, Decision-making, Distributive preferences, Computational modeling, Psychology, Human behaviour

## Abstract

Wealth inequality is one of the most profound challenges confronting society today. However, an important issue in addressing inequality lies in formalizing the diversity of *individual* perspectives regarding what constitutes a fair distribution of resources. We tackle this topic by simulating wealth inequality through the allocation of bonus endowments in both Dictator Game (DG) and Ultimatum Game (UG) settings and capturing distributive decisions. By integrating a computational model, we quantify individual differences in the interplay between financial self-interest and competing pro-social motivations that emerge in the context of pre-existing wealth inequity. Our behavioral results show that, on average, pre-existing wealth influences distributive preferences across both allocations and proposals. Yet, inequality elicits non-uniform fairness concerns. Using a hierarchical clustering approach, we objectively categorise participants’ behavior elucidating four distinct decision strategies: ‘Pro-Self’, ‘Table Egalitarianism’, ‘Total Egalitarianism’, and ‘Moral Opportunism’. A balanced distribution of strategies is observed during allocations (DG), whereas Table Egalitarianism prevails in strategic proposals (UG), highlighting the influence of strategic considerations on decision strategy. Furthermore, we demonstrate an association between strategies across decision contexts. Our findings thus contribute a principled framework to formalize distributive preferences, revealing that, with respect to both altruistic allocations and strategic proposals, competing ideals of fairness underlie distributive preferences under wealth inequality.

**Supplementary Information:**

The online version contains supplementary material available at 10.1038/s41598-024-83361-z.

## Introduction

Wealth distribution is strikingly unequal. Globally, the top 10% of the population owns 76% of total worldwide wealth, while the bottom 50% owns a meager 2%^1^. As this uneven division between rich and poor persists^2^, one of the most pressing societal questions is how to achieve an equitable distribution of resources in light of existing wealth disparities. Yet, proposals for resource distribution trigger heated discourse about concepts of fairness and equality^3^. As evidenced by extensive debate across political, economic, legal, sociological and psychological perspectives, opinions of what constitutes a ‘just’ allocation frequently diverge^4–9^. Should individuals who possess less receive a greater share of resources? Is it fair for people with more than others to forfeit their claim to valuable commodities? Questions about the prevailing consensus regarding distributive preferences, and whether people consistently adhere to their notions of fairness, have endured as a central theme across history, while also underscoring current discussions in 21st century politics. Here, we provide a precise analysis of individual distributive preferences in situations of pre-existing wealth inequality. We do so by computationally characterizing preferences towards resource distribution, offering conceptual clarity on how decision strategies vary among individuals and across contexts.

The impact of existing wealth on distributive choice can simply be illustrated through a scenario inquiring how a shared restaurant bill should be split between two diners. Assuming that both owe an equal amount, some would contend that the fairest approach is a 50–50 split. However, if one of the diners possesses significantly more wealth than the other, some might advocate for considering this broader economic context, suggesting that the richer individual should assume greater responsibility for the bill, if not covering it entirely. This illustrative scenario serves as an example of resource distribution, and outlines the competing ideals involved when individuals with different wealth profiles interact. Furthermore, this example draws clear parallels to debates regarding the construction of laws and social welfare policies (such as taxation and the distribution of health or educational resources), where concerns arise that ignoring wealth may, directly or inadvertently, exacerbate existing inequality^10,11^. Concurrently, critics (particularly those falling along conservative political lines in Western societies) argue that going after the wealthiest in society would in fact not effectively benefit those at the lower end of the economic spectrum^12^. As a result, the societally important topic of resource allocation often culminates in polarizing political opinions and legal standoffs^13^.

In situations like the above scenario, and in policy discussions in general, a critical question is what exactly constitutes a fair distribution^5^. In this regard, economists, psychologists, and sociologists have long recognized that individuals are strongly motivated by fairness considerations and are generally averse to inequitable outcomes^11,14^. Specifically, extensive research has compellingly demonstrated that people frequently make decisions that do not align with the sole goal of maximizing their own monetary earnings^14–18^. Models of inequality aversion, including the seminal works of Fehr and Schmidt^18^ and Bolton and Ockenfels^15^, explicitly attempt to quantify people’s distributive fairness preferences relying on the assumption that the utility of a decision outcome depends on both one’s own payoff, as well as how this payoff compares to that of others. When applied to well-established allocation tasks such as the Dictator Game (DG), where a decision-maker unilaterally decides how to divide an endowment, and the Ultimatum Game (UG), where a game partner has the power to punish a decision-maker by rejecting their proposal, inequality aversion models empirically describe both selfish and prosocial findings in situations of altruistic allocations and strategic bargaining^15^. A crucial aspect of these models, hitherto left underexplored, is that individuals might differ in the weighting they attach to fairness considerations relative to their own economic self-interest^5^. Indeed, empirical findings have documented considerable heterogeneity in the relative weight people attach to fairness in both games: some participants choose to take everything for themselves, some divide equally, and some choose intermediate distributions^5,19^.

Nonetheless, ongoing research suggests that inequality aversion alone may not offer a comprehensive explanation of human behavior in scenarios involving resource distribution^3^. The observation that people view some inequalities as more fair than others has spurred an increasing interest into the contextual factors which individuals consider when making judgments^5^. Of particular relevance here, several prior studies have examined the impact of relative wealth on fairness considerations, though yielding rather mixed results to date^20–27^. Liebe et al.^28^ found that across four countries individuals with a higher socioeconomic status (SES) tend to be more altruistic in monetary distributions in the DG, and the recipient’s SES has a significant influence on monetary donations. Similarly, Smeets et al.^29^ observed that real-world millionaires gave, on average, half of their experimental endowment to other millionaires in both DG and UG. However, when interacting with individuals of average SES, they offered a much more generous allocation in both games. Interestingly, there were substantial individual differences observed in this study, with approximately 45% of the millionaires offering the full endowment in the DG, while roughly 20% offered only a half, suggesting that these latter group of millionaires disregarded existing wealth differences and only considered the task stakes, in line with standard inequality aversion models^18,29^. Overall, the (limited) studies examining the impact of pre-existing wealth disparities indicate that these differences have varying degrees of influence in decisions about the distribution of money. An important challenge for understanding fairness in distributive preferences thus lies in addressing and formalizing the diversity of perspectives^5^. Therefore, one goal of this research is to computationally quantify individual differences in distributive preferences.

Building on the divergence in preferences observed through empirical data, as well as political and legal standpoints, two competing ideals of fairness in respect of wealth inequality emerge. On one side is the perspective that focuses on addressing immediate equality only, while the other pursues a broader context that encompasses pre-existing inequalities. When these concepts are integrated into an inequality aversion model, they provide a clear conceptual framework for exploring distributive behaviors in situations of varying wealth disparities. Accordingly, in scenarios where individuals distribute resources among those with differing wealth levels, we propose that three key factors come into play: self-interest motivations and two distinctive ideals of fairness.

Self-interest is, of course, self-evident: we define this as the choice made to increase the benefit of the decision-maker, without consideration of the other. The first fairness ideal, which we term ‘Table Egalitarianism’, draws inspiration from poker, where ‘table stakes’ limit the amount a player can bet on a particular hand. In this context, wealth that players possess beyond these stakes is irrelevant for the current round of betting. Consequently, Table Egalitarianism predicts that when making distributive choices, individuals only consider addressing inequalities within the immediate game context (i.e. the monetary endowment available for distribution). That is, in the context of DG and UG, the Distributor may be prepared to share the endowment, but will not use it to address pre-existing disparities, either advantageous or disadvantageous. The second, competing fairness ideal we propose is ‘Total Egalitarianism’. This principle states that individuals aim to minimize all inequalities between players, including those related to pre-existing wealth in addition to those arising from the game context. Therefore, this ideal predicts that an individual will consider overall wealth differences when deciding how to distribute resources, and will use these resources to balance the total inequality between the players. If the Distributor has less overall wealth than the Receiver, then they will keep more of the endowment. However, if the Distributor is wealthier than the Receiver, they would be willing to part with more of the resource.

**Fig. 1:**
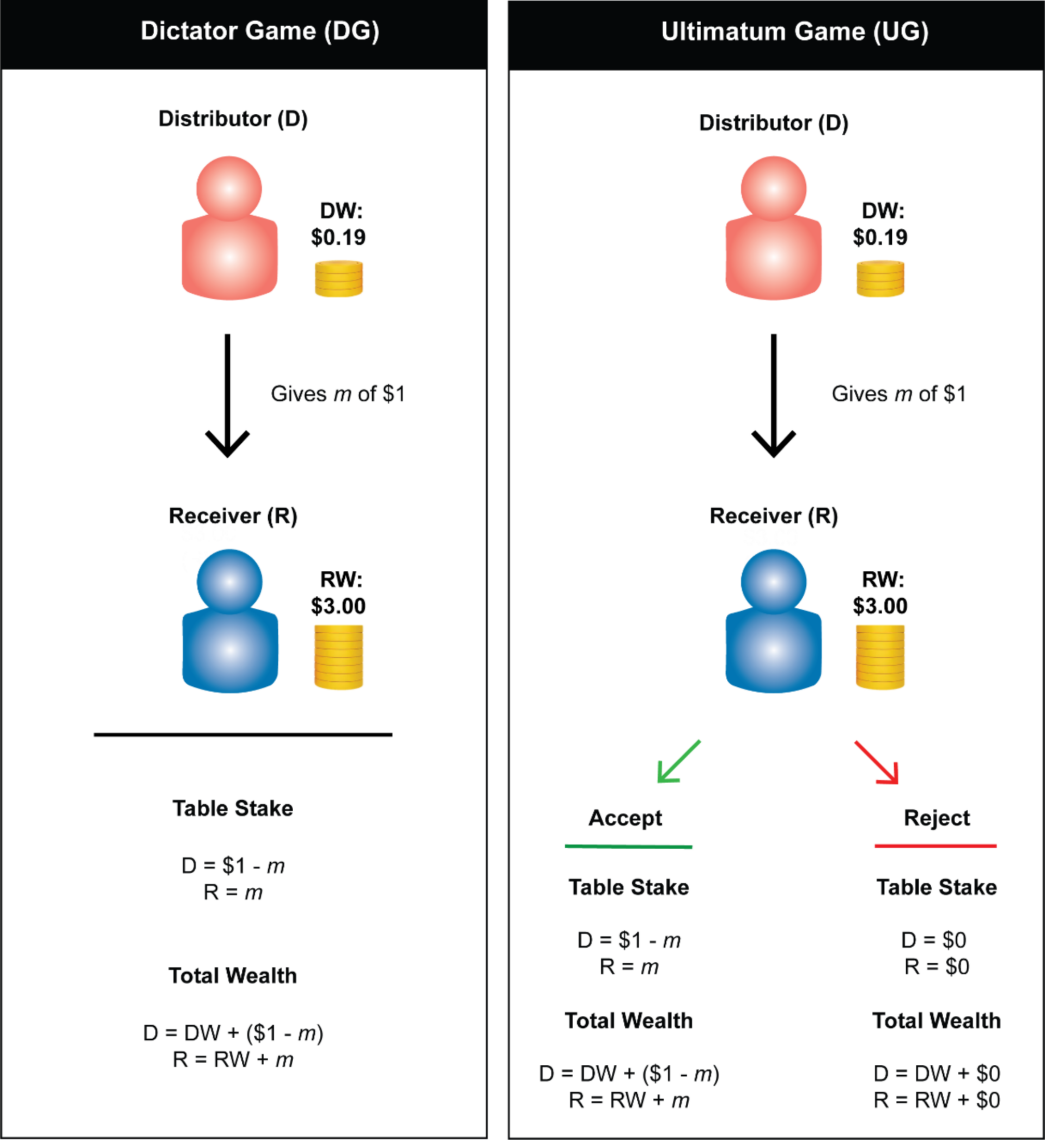
In both the Dictator Game (DG, left) and the Ultimatum Game (UG, right), there are two players: one is assigned the role of the Distributor (D) and the other assigned as the Receiver (R). At the start of the game, both players get a bonus referred to as Distributor Wealth (DW) or Receiver Wealth (RW) respectively. Thereafter, D is endowed with $1 while R gets nothing ($0). In the DG: D is given the option to give a share of the $1 endowment (m) to R; R passively accepts. In the UG: The rules are similar to the DG, except R is now allowed to accept or reject the choice of D. If R accepts, the split is allocated per D’s offer, if R rejects, both players receive nothing.

The central question in the present study is to explore whether, and if so how, people employ these two proposed norms in distributive decision-making (Fig. 1 provides a schematic overview of the task). To do this, in a within-subjects behavioral experiment we provide participants in an online study with varying levels of pre-existing ‘wealth’ and then investigate preferences to share an unrelated endowment within the context of the DG, which are pure allocation choices. We also examine whether these fairness ideals exhibit sensitivity to the strategic aspects of a decision by analyzing choices in the UG. We assess the degree to which participants adhere to the strategies outlined above, or demonstrate a mix of strategies as a function of the specific game context.

As the predominant behavior in the UG is to give up approximately half of the endowment^30^, we hypothesize that within our sample Table Egalitarianism will be the most prevalent strategy in this decision context. Moreover, in line with previous findings^29^, we anticipate observing higher self-interest in DG decision behavior. Nonetheless, prior work has highlighted strategic distinctions between the DG and UG which can impact decision behavior across games^31^. As a result, we hypothesize that people who exhibit self-interest in the DG, might tactically transition toward a more prosocial decision strategy in the UG. Specifically, we expect that selfish behavior in the DG will correlate with an increased inclination towards Table Egalitarianism in the UG. On the other hand, research has shown that prosocial behavior tends to be consistent across domains and contexts^19,32^. In line with this, we predict that people who behave in a prosocial manner in the DG, do not feel the need to adapt their behavior in the UG. In the specific context of our proposed fairness ideals, we hypothesize that when a person exhibits Total Egalitarian behavior in the DG, they will adhere to the same strategy in the UG. Similarly, when a person adopts Table Egalitarian behavior in the DG, we anticipate that they will be inclined to follow the Table Egalitarian ideal in the UG.

To provide a structured framework to answer these questions and allow a more nuanced examination of individual differences in preference weighting, we develop a computational model adapted from the traditional inequality aversion model^18^. This model allows us to examine how participants’ make decisions, navigating trade-offs between Total and Table Egalitarian distributions, in addition with economic self-interest, thereby connecting observed behaviors with theoretical principles of inequality aversion and distributive justice. Capturing behavior governed by the two prosocial rules, Table and Total Egalitarianism, also allows us to investigate the possible occurrence of Moral Opportunism^33,34^. As observed in prior empirical work, this preference captures participants who essentially choose a pro-social strategy, but select the specific one that maximizes their personal financial gain^33,34^.

By integrating a computational model with behavioral data from our controlled experiment, we help clarify how pre-existing wealth differences shape decision-making processes during allocations and proposals, at an individual level. Specifically, for both decision contexts studied here—the DG and UG—we employ our utility model, termed the Two Norms model (Eq. 1), to characterize distributive strategies. This model incorporates two free parameters (φ and ϑ) that capture different sources of utility—Pro-Self (φ), Table Egalitarianism, and Total Egalitarianism reflecting the trade-offs participants navigate during decision-making:

1 $${{\text{U}}_{({\text{Two Norms}})}}\,=\,{\text{}\phi}*{\text{ }}{{\text{m}}^{\text{D}}} - {\text{ }}({\text{1 }} - {\text{}\phi}){\text{ }}*{\text{ }}({\text{}\theta}*{\text{ }}{{\text{I}}_{{\text{Table}}}}+{\text{ }}({\text{1 }} - {\text{}\theta}){\text{ }}*{\text{ }}{{\text{I}}_{{\text{Total}}}})$$

Here, m^D^ reflects the amount an individual chooses to keep for themselves during allocations (DG) or proposals (UG). I_Table_ represents the ideal of Table Egalitarianism, which seeks to reduce inequalities stemming solely from the Table Stake (namely, the experiment endowment). This is achieved by dividing the endowment equally, disregarding any pre-existing wealth disparities. In contrast, I_Total_ captures the principle of Total Egalitarianism, which aims to reduce total inequality, including the pre-existing wealth differences between parties. This is accomplished by using the Table Stake to minimize existing wealth disparities.

In order to objectively analyze the presence of different motivations underlying decisions, namely Moral Opportunism, as well as Total Egalitarianism, Table Egalitarianism, and Pro-Self preferences, we implement hierarchical clustering allowing us to identify participant behavior, systematically shedding light on distinct decision strategies that underlie distributive preferences. A comprehensive description of the experimental setup, computational model, and clustering approach is provided in the “Methods” section.

## Results

### Allocation and proposal decisions

#### Dictator game

**Fig. 2:**
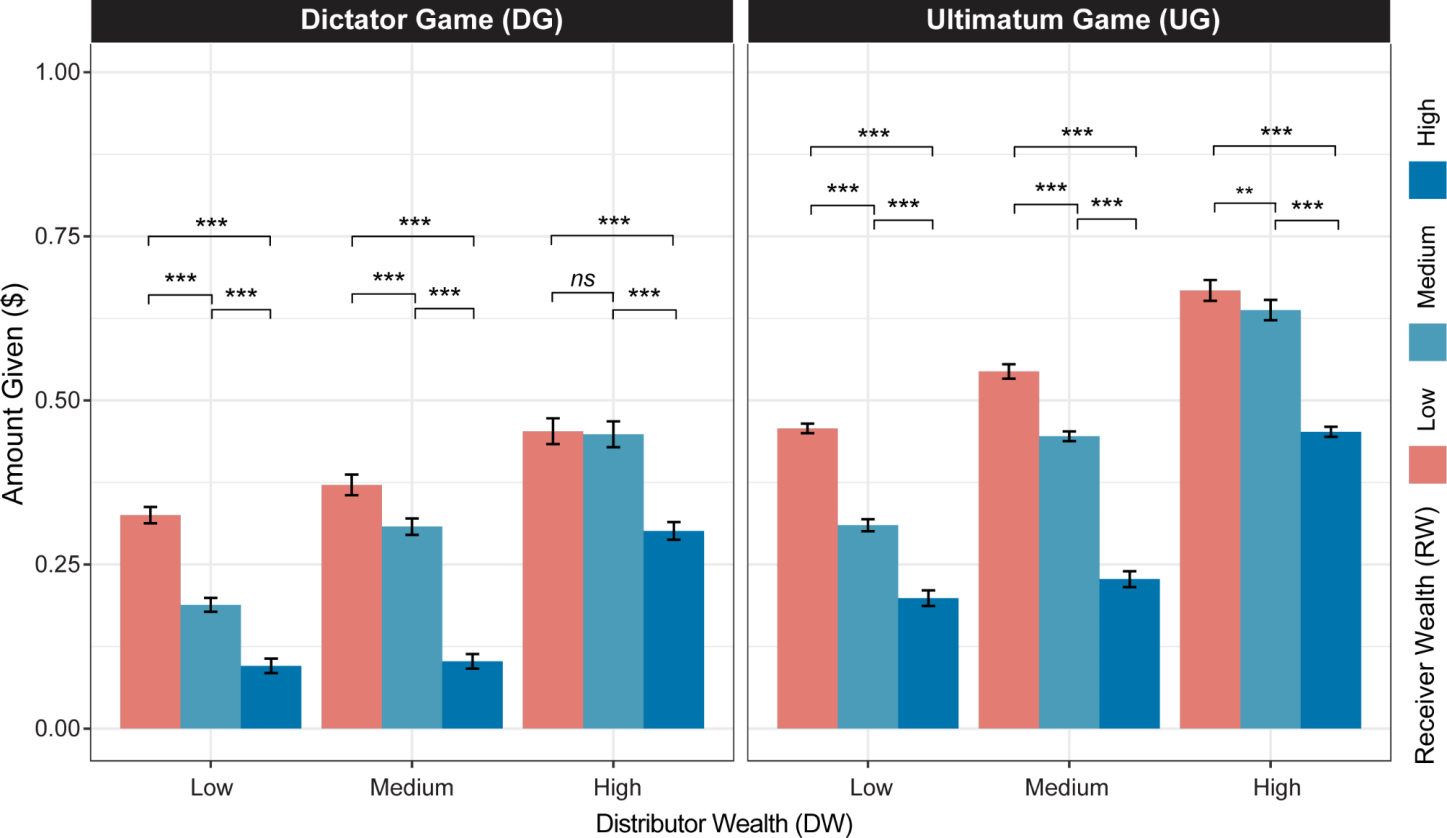
Mean amount given ($) for each distributor wealth (DW) (x-axis) and receiver wealth (RW) condition in Dictator Game (DG) (left) and Ultimatum Game (UG) (right). For both DW and RW, “Low” = $0.19, “Medium” = $0.75, and “High” = $3.00. Error bars reflect standard error of the mean.

On average, participants in the DG gave $0.28 (± $0.29) of the endowment to the Receiver. A repeated-measures ANOVA was conducted to examine the effects of two within-subject factors on participants’ distributive choices in the DG, namely wealth belonging to the participant (DW) and wealth belonging to the Receiver (RW) (Fig. 2). The results revealed a significant main effect of DW (*F*(2, 214) = 54.95, *p* < 0.001, *η*^2^ = 0.1), indicating that participants’ choices varied as a function of their own wealth level: the more wealth a Distributor had, the more they choose to give to the Receiver. A significant main effect of RW was similarly observed (*F*(2, 214) = 95.69, *p* < 0.001, *η*^2^ = 0.12), indicating that, on average, a Receiver’s wealth level influenced how much they received. This effect is best understood in light of a significant interaction between DW and RW (*F*(4, 428) = 16.74, *p* < 0.001, *η²* = 0.01), indicating that the influence of RW on distributive decisions depended on the Distributor’s wealth level, as explained more fully in post-hoc analysis.

More specifically, post-hoc analysis revealed that when Distributors had equal wealth to the Receivers (e.g. DW = RW = $0.19 or $0.75 or $3.00), giving behavior was not significantly affected by the wealth endowments (*p* = 0.90). Specifically, when there were no pre-existing differences between wealth levels of the Distributor and Receiver, the Distributor chose to give on average $0.31 (± $0.40). This contrasts situations involving wealth inequality. In particular, when participants had the lowest wealth level ($0.19) and were paired with Receivers having the highest wealth ($3.00), participant’s chose to give on average $0.10 (± $0.20). However, when Distributors were in the highest wealth condition ($3.00), they gave far more—approximately $0.45 (± $0.35)—to Receivers in the lowest wealth condition ($0.19). Furthermore, pairwise comparisons suggests differences in sensitivity to wealth in the DG which varies depending on condition. When a low wealth participant ($0.19) interacts with a high wealth participant ($3.00), a slightly greater effect size is observed (*Estimate* = 0.23, SE = 0.02, *t-ratio* = 10.30, *p* < 0.0001), compared to situations where a high wealth participant ($3.00) interacts with a low wealth participant ($0.19) (*Estimate* = 0.15, SE = 0.02, *t-ratio* = 6.85, *p* < 0.0001). In both cases, the interactions are compared with those involving participants interacting with Receivers of the same wealth condition. Table S1 shows pairwise comparisons conducted for each of the levels and pairs of DW and RW in the DG. Overall, these results suggest that in the DG, both the initial wealth levels of Distributors and Receivers influence how the $1 endowment is split.

#### Ultimatum game

On average, participants in the UG gave $0.44 (± $0.26) of the endowment to the Receiver. A repeated-measures ANOVA was conducted to examine the effects of two within-subject factors on participants’ distributive choices in the UG, namely wealth belonging to the participant (DW) and wealth belonging to the Receiver (RW) (Fig. 2). The results showed that there was a significant main effect of Distributor wealth (*F*(2,214) = 131.77, *p* < 0.001, *η*^2^ = 0.26) and of Receiver wealth (*F*(2,214) = 151.93, *p* < 0.001, *η*^2^ = 0.25) respectively on distributive choices. Additionally, the interaction between DW and RW was significant (*F*(4,428) = 21.66, *p* < 0.001, *η²* = 0.02), indicating that the effect of the Distributor’s wealth on how the endowment was split depended on the level of the Receiver’s wealth.

As in the DG, post-hoc analysis revealed a distinction in participants’ giving behavior in the UG based on the wealth levels of Distributors and Receivers respectively. When Distributors were paired with Receivers of equal wealth (DW = RW = $0.19 or $0.75 or $3.00), there was no significant impact of wealth on giving behavior (*p* = 0.10). More specifically, in conditions of wealth equality, participants gave on average $0.45 of their endowment. This is in contrast to conditions of wealth inequality. Specifically, when Distributors with the lowest wealth level ($0.19) were paired with Receivers with the highest wealth ($3.00), average giving behavior was approximately $0.20 (± $0.21). The opposite is true when Distributors had the highest level of wealth ($3.00), and Receivers had the lowest ($0.19), with mean giving behavior increasing to $0.67 (± $0.28). Additionally, pairwise comparisons suggests differences in sensitivity to wealth in the UG which vary depending on condition. When a low wealth participant ($0.19) interacts with a high wealth participant ($3.00), a slightly greater effect size is observed (*Estimate* = 0.26, SE = 0.02, *t-ratio* = 13.77, *p* < 0.0001), compared to situations where a high wealth participant ($3.00) interacts with a low wealth participant ($0.19) (*Estimate* = 0.22, SE = 0.02, *t-ratio* = 10.33, *p* < 0.0001). In both cases, the interactions are compared with those involving participants interacting with Receivers of the same wealth condition. Table S2 shows pairwise comparisons conducted for each DW and RW pair in the UG. These results thus suggest that the initial wealth levels of Distributors and Receivers influence participants’ choices in the UG and that the interaction of these effects significantly impacts participants’ choices.

#### Dictator game versus ultimatum game

In line with previous literature (Camerer, 2011), participants gave more money in the UG ($0.44 ± $0.26) relative to the DG ($0.28 ± $0.29) (F(1, 107) = 82.73, *p* < 0.001, η^2^ = 0.105). A repeated-measures ANOVA showed significant main effects for DW, RW, and game, as well as a significant interaction between DW and RW (all ps < 0.001). Specifically, Distributors offered greater amounts of the $1 endowment when they had more wealth (F(2, 214) = 110.31, *p* < 0.001, η^2^ = 0.164) or when the Receiver had less wealth (F(2, 214) = 147.46, *p* < 0.001, η^2^ = 0.173). The interaction between wealth of the Distributor and wealth of the Receiver was also significant, indicating that the effect of wealth of participant on distributive choice varied depending on the level of wealth of the other player (F(4, 428) = 30.05, *p* < 0.001, η^2^ = 0.015). The interaction between DW, RW and game played was, however, not significant (*p* > 0.05). These findings thus suggest that participants’ choices were influenced by both the participant’s own wealth and the wealth of the Receiver, as well as the game context.

### Computational model

#### Model fit and comparison

After fitting the Total, Table and Two Norms models for each participant for each of the games separately, we computed Akaike Information Criterion (AICs) and Bayesian Information Criterion (BICs) across models. The ‘Two Norms’ model exhibited lowest mean AIC and BIC values compared to the other two models in both the DG (M_AIC_ = − 238.55; M_BIC_ = − 235.96) and UG (M_AIC_ = − 244.47; M_BIC_ = − 241.90). In order to determine if the ‘Two Norms’ model provides the best fit among the three models, we conducted Wilcoxon signed-rank tests on subject-wise BIC as well as AIC differences for both the DG and UG respectively.

In respect of BIC values, and for the DG, the tests revealed the Two Norms model was a better fit relative to both Table (*n* = 108, V = 723, and *p* < 0.001) and Total (*n* = 108, V = 557, *p* < 0.001) models respectively, with no statistical difference between Table and Total models (*p* = 0.63). Similarly, for the UG, the tests revealed a significant difference between the Two Norms and Table (*n* = 108, V = 406, *p* < 0.001), and Two Norms and Total (*n* = 108, V = 114, *p* < 0.001), but no difference between Table and Total models (*p* = 0.07).

In respect of AIC values, for the DG, Wilcoxon signed-rank tests indicated significant differences in AIC values when comparing the Two Norms model with both Table (*V* = 624, *p* < 0.001) and Total (*V* = 496, *p* < 0.001), with no difference between Table and Total models (*p* = 0.63). In the UG, there were similarly pronounced differences between the Two Norms model and both Table (*V* = 405, *p* < 0.001) and Total (*V* = 78, *p* < 0.001), and no significant difference between the Table and Total models (*p* = 0.07).

Accordingly, for both the DG and the UG, the Two Norms model was a significantly better fit to the data as compared to the Table and Total models respectively (see: Table S3 for an overview of model parameters and fit per model for DG and UG; Fig. S2).

### Individual variation in decision strategy

Next, we examined the decision strategies via the hierarchical clustering approach. Specifically, we sought to explore the frequency of participants that followed the different motives in the DG and UG and the relationship between φ and ϑ parameters in each game.

Our analysis revealed distinct strategic preferences in the DG and UG. Specifically, in the DG, the predominant strategy was Pro-Self (28.70%) followed by Total Egalitarianism (26.85%), Table Egalitarianism (25.93%) and Moral Opportunism (18.52%). Conversely, in the UG, Table Egalitarianism emerged as the dominant strategy (52.78%), followed by Total Egalitarianism (31.48%), Moral Opportunism (10.19%) and Pro-Self (5.56%). These differences in strategy based on decision context were confirmed through Pearson’s chi-squared test, which demonstrated a significant influence of the game played on participant’s clustering (χ^2^(3) = 29.80, *p* < 0.001).

**Fig. 3:**
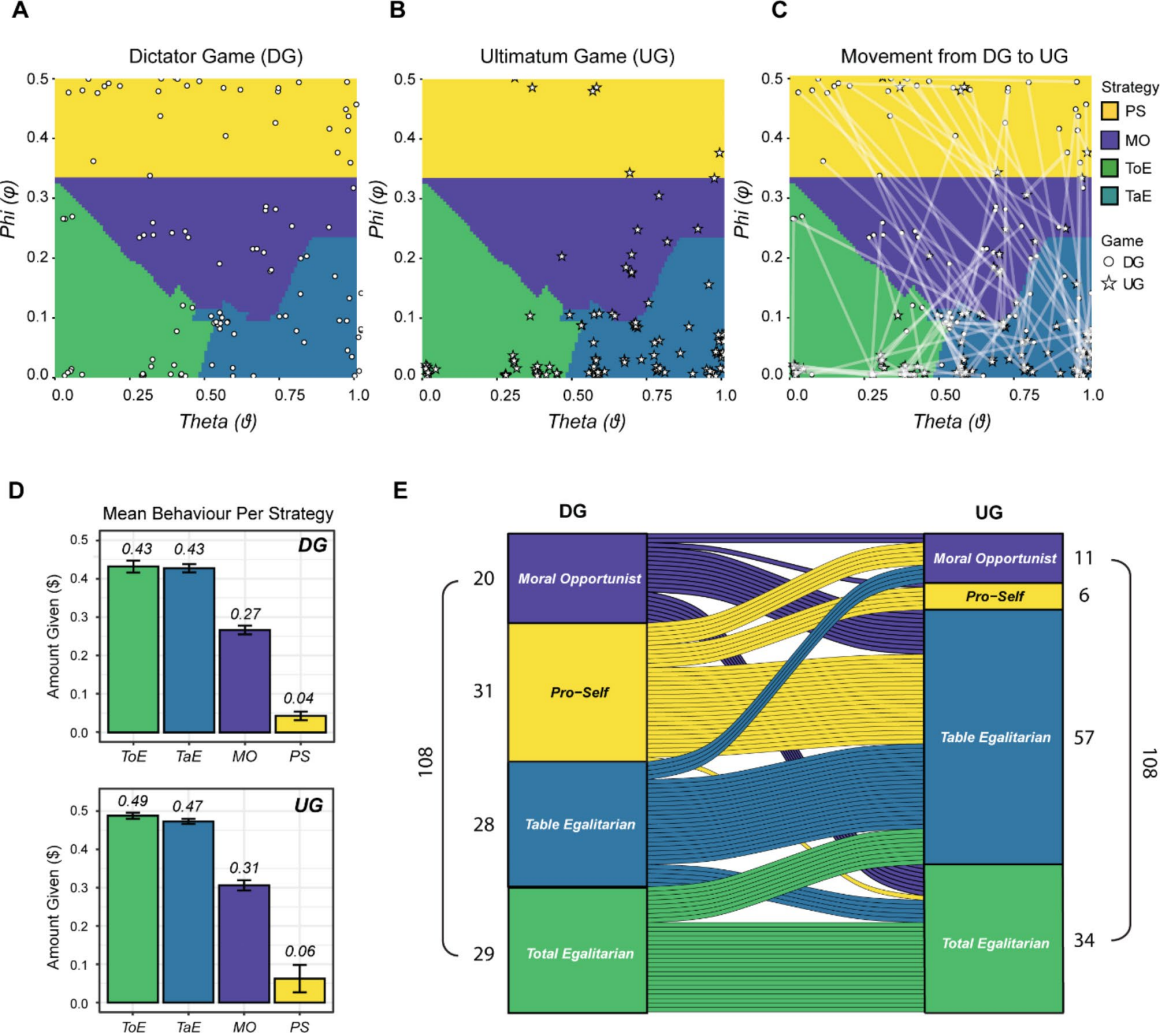
**(A–C)** 2D theta-phi decision space, where distinct strategies are delineated by cluster surface colors. The behavior of individual subjects in the (A) Dictator Game (DG) and the (B) Ultimatum Game (UG) are represented by white dots and white stars respectively. (C) Two data points per individual, one for the strategy in the DG and one for the strategy in the UG. Data points belonging to the same participant are connected by a white line. (D) Mean giving behavior per strategy group in the DG (top) and UG (below). Error bars represent standard error of the mean. (E) River plot illustrating the frequency and movement between decision strategies from the DG to UG.

Additionally, we employed Spearman’s rank correlation to explore the relationship between φ and ϑ in both the DG and UG. We observed no correlation between parameters in the DG (*rho* = 0.0334, *p* = 0.73), compared to a weak positive correlation in the UG (*rho* = 0.3345, *p* < 0.001). These findings illustrate differences in the relationship between parameters, with a more pronounced association in the UG compared to the DG, highlighting the impact of strategic consideration on these correlations. Figure 3 illustrates individual strategies as a function of φ and ϑ coordinates in DG (*3A*) and UG (*3B*).

As confirmatory analysis of cluster differences, we investigated mean behavior across decision strategies (see Fig. S3). Welch’s ANOVA revealed significant differences in giving behavior across the four clusters (F(3, 2528.8) = 2195.5, *p* < 0.001) (Fig. 3D). Pro-Self participants gave away the lowest amount of the endowment across both games (M_DGandUG_ = $0.05; M_DG_ = $0.04; M_UG_ = $0.06). This was significantly different from behavior in the three other strategy groups (all *p’s* < 0.001). Participants who followed the Moral Opportunist strategy gave away more than those in the Pro-Self cluster (M_DGandUG_ = $0.28; M_DG_ = $0.27; M_UG_ = $0.31, *p* < 0.001), but less than both Table and Total Egalitarians (both *p’s* < 0.001). Table and Total Egalitarians did not differ significantly, with each group giving away the same approximate mean amount of $0.46 (*p* = 0.67).

### Stability of decision strategies across contexts

To further understand the stability of cluster groups between DG and UG, we examined movement between strategies (Fig. 3C). Within each cluster, pairwise chi-squared tests revealed a statistically higher frequency of the Pro-Self strategy in the DG (31 participants) compared to the UG (6 participants) (χ^2^(1) = 16.90, *p* < 0.001). Moreover, there was significantly higher number of participants who followed the Table Egalitarian motivation in the UG (57 participants) compared to the DG (28 participants) (*χ*^2^(1) = 9.90, *p =* 0.002). While there were more individuals who adopted the Total Egalitarian strategy in the UG compared to the DG, this difference was not statistically significant (*p* = 0.53). Similarly, there were fewer Moral Opportunists in the UG compared to the DG, although this difference was also not statistically significant (*p* = 0.11). Furthermore, among those participants who remained in the same decision cluster between games, 19.44% consistently adhered to the Total Egalitarian strategy, while 17.59% showed a persistent preference for Table Egalitarianism. In contrast, 18.52% (within the Pro-Self group) and 9.26% (within the Moral Opportunist group) of participants in the DG changed strategy, transitioning to the Table Egalitarian strategy in the UG (Fig. 3E).

Importantly, we found evidence that changing motivation between games was associated with the strategy adopted in the DG. Specifically, we conducted Spearman’s correlations for ϑ between DG and UG revealing a strong and statistically significant positive correlation between games (*rho* = 0.5111, *p* < 0.001), highlighting that the social decision strategy adopted in the DG strongly aligns with the social decision strategy adopted in the UG. Accordingly, individuals who exhibited prosocial behavior in the DG maintained their social preferences consistently across games.

### Socio-demographic variables

As exploratory analyses, we investigated the relationship between the combined φ and ϑ parameters and the socio-demographic variables of the participants via MANOVA analyses of DG and UG separately. In the DG, religion (*p* = 0.003), self-reported personal income (*p* = 0.004), age (*p* = 0.03) and political orientation (*p* = 0.05) emerged as significant contributors in shaping participants’ allocations in the DG. Conversely, education, family income, household income, and subjective relative SES were not of statistical significance. In the UG, none of the demographics analysed were found to be significant predictors of the model parameters. These differences thus suggest a nuanced relationship between the socio-demographic factors and distributive decisions in the DG compared to the UG. (see: Fig. S4; Table S4).

## Discussion

The significant wealth gap between rich and poor has prompted extensive research across diverse fields attempting to better understand the impact of wealth differences on individual decision-making^2,5^. Influential theories have shown that people are generally averse to inequality and are willing to make trade-offs between their own self-interest and that of others, helping to explain distributive behavior in established economic games like the Dictator Game (DG) and Ultimatum Game (UG)^15–18^. However, to the best of our knowledge, most models of inequality aversion do not account for an important contextual factor namely, knowledge of the wealth-related similarities or differences of a counterpart. This raises the possibility that conventional explanations of decision behavior in these games (and their associated models) may overlook a crucial aspect inherent to real-life social exchanges. Additionally, research examining the extent to which wealth plays a role in shaping individual notions of fairness have to date produced conflicting results about the impact of wealth disparities on distributive decision-making^29,35^. These inconsistencies highlight the need for a structured and principled understanding at the individual level as to how people approach the question of how to divide resources in situations of wealth disparities.

Our study advances existing research on distributive preferences by establishing a controlled experimental paradigm which simulates wealth inequality and then investigating the extent to which wealth plays a role in allocation and proposal decisions. Furthermore, drawing inspiration from inequality aversion models^15,18^, and bridging opposing ideals of fairness evidenced through legal and political opinions^12,13^, we develop and integrate a computational model to explore the interplay between self-interest and considerations of pre-existing wealth in distributive choices, at the individual level. Our results indicate that competing fairness ideals emerge in situations of even small wealth inequality, and that the model which best captures how participants make distributive choices is one which estimates their selfish tendencies and the degree to which they consider pre-existing wealth status. Our hierarchical clustering approach offers a systematic framework for understanding distinct decision strategies that help to understand variations in behavior observed across individuals and across contexts.

Average giving behavior in our study replicates previous findings^30^. Specifically, participants offered 28% of the endowment in their allocation decisions (DG), which was significantly lower than the 44% in their proposal choices (UG). These differences highlight both the importance of the game context in participants’ choices, and also serve to confirm that our online sample behaved similarly to participants in previous laboratory settings^36^.

Importantly, our behavioral results clearly demonstrate an influence of wealth inequality on participants’ giving behavior across both games, which appears even in light of the relatively small wealth differences between the players. When both Distributor and Receiver possessed an equal bonus amount, their level of wealth did not impact giving behavior. However, in situations of wealth inequality, on average, participants adjusted their giving behavior according to their respective wealth level. Specifically, and in line with previous findings in the literature^26,29^, we observed that wealthier participants gave away relatively more money to those who had less, and less wealthy participants kept relatively more money when interacting with those who possessed more. In the UG in particular, wealthier participants on average exhibited a greater willingness to give away more money and were particularly inclined to do so when Receivers possessed a lower wealth level. Furthermore, our findings suggest a potential asymmetry in sensitivity to wealth. Participants tended to be more sensitive to wealth when they were in the low compared to high wealth group, highlighting a possible heightened awareness of wealth disparities when experiencing low wealth levels. It is important to note that participants in our study were never instructed, either implicitly or explicitly, to take account of pre-existing differences in their DG/UG decisions, but this behavior emerged naturally from the context. Together, our behavioral results thus contribute a valuable insight into participants’ sensitivity to wealth-based disparities, showing the influence of both Distributor and Receiver wealth in distributive choices across settings.

Studying giving behavior with a modelling approach allows us to shed a more nuanced light on the decisions in our tasks helping to uncover differences among individuals. Building upon prior work, our modified inequality aversion model elucidates diverging fairness ideals at an individual level in order to probe, in a principled manner, different distributive preferences that may exist in our sample^18,34^. In our model, fairness preferences are defined across two dimensions: a self-interest axis that ranges from altruistic to selfish (φ), and a dimension that ranges between two different types of prosocial strategies, Total to Table Egalitarianism (ϑ). This latter parameter essentially captures the extent to which players take inequalities unrelated to the immediate game setting into account. We estimated the two free parameters per Distributor per game, allowing us to draw meaningful inferences about underlying preferences of behavior which are not readily discernable through simply averaging giving behavior. Our model outperformed two alternative models which respectively controlled for only one of the fairness norms, indicating that a significant portion of our sample employed either Table and Total Egalitarian ideals in each game.

We further employed hierarchical clustering to objectively categorize distinct preferences^34^, identifying the presence of four dominant decision strategies. “Pro-Self” reflects individuals who seek to maximize their own profits by making choices that consistently prioritize only their own economic self-interest. “Table Egalitarianism” represents a decision-making approach where individuals focus on achieving an equal split of the table stake disregarding any existing wealth inequalities. “Total Egalitarianism” captures participants who use the table stake to equalize all inequalities, including those created by the wealth disparities between players. Lastly, “Moral Opportunism” is a hybrid decision strategy combining elements of both Total and Table Egalitarianism^33,34,37^—here the player selects the (prosocial) option that results in the least financial cost in each specific situation. Importantly, the choice between Total and Table Egalitarianism is not random for those practicing Moral Opportunism, but is rather guided by a preference for the financially less expensive option in each given context^34^. Accordingly, we report that multiple possible strategies could be applied by Distributors in situations of varying wealth inequality, resulting in variations in mean giving behavior within and between DG and UG.

A critical contribution of our study is revealing that participants approach distributive choices using different strategies. In the DG, we find a relatively even distribution between the four strategies. While there were a slightly greater number of participants who made allocation decisions to maximize their own financial benefit, similar numbers of players focused their attention on only the game stakes or on the overall wealth differences. Interestingly, these Table and Total Egalitarians exhibited the same mean giving behavior, however our approach illuminates the fact that these two groups differ in their decision-making in an important way, namely their notably different concern for pre-existing wealth differences. In the context of the UG, a dominant strategy emerged with more than half of all participants converging on Table Egalitarianism, followed by approximately one third who adopted Total Egalitarianism as their strategy. The relationship between self-interest and prosocial tendencies differed between the two games, and we show that the specific strategic considerations of the decision context do influence decisions made by the Distributor. Accordingly, a clear advantage of the methodological approach employed here lies in its ability to capture these important individual dynamics underlying distributive preferences, which are difficult to discern and formally capture by examining only average group behavior.

Notably, our cross-game analyses show that while some players are consistent in their decision strategy, others adapt their approach based on the distinct context presented by the DG and UG. Specifically, the majority of participants who followed the Pro-Self and Moral Opportunist strategy in the DG switched to Table Egalitarianism in the UG. As the two former strategies are defined by relatively high self-interest, we propose that this shift highlights the influence of these individuals’ pro-self tendencies, driven by strategic considerations, on their decision to change approach. In particular, the inclination of these players to offer increased amounts in the UG may reflect an adjustment of strategy aimed at maximizing the likelihood of offer acceptance, making their behavior resemble egalitarian despite remaining fundamentally self-interested. As the uncertainty inherent in the Receiver’s response in the UG is not expressly accounted for in our model, future extensions of the model could explore this more fully by incorporating Receiver decision-making, such as the possibility of offer rejection. These extensions would help provide a more comprehensive understanding of the role of strategic considerations in distributive behavior under wealth inequality in the UG specifically.

On the other hand, the Table and Total Egalitarian strategies in the DG were associated with use of the same strategies in the UG. This finding suggests that when participants behave in a prosocial manner, they follow a similar norm across both of our decision settings. One possible explanation for such stability lies in the abundance of research which has found intraindividual prosocial consistency within and across games^19,30,35,38^. Moreover, our findings are broadly consistent with the idea of a stable prosocial or ‘cooperative phenotype’^32^. In this regard, we show the methodological potential of mapping the trajectory of strategies in revealing consistent preferences across decision contexts.

The occurrence of competing fairness ideals raises several interesting questions. Further studies could usefully explore *why* participants adopted either Table or Total Egalitarian motivations. It’s plausible that participants may have perceived the wealth allocation in our task as fair, leading them to follow Table Egalitarianism. Conversely, if they interpreted the procedure as unfair, they may have opted for a Total Egalitarian strategy. This reasoning aligns with the observation that individual differences exist in people’s perceptions of fairness regarding distributions based on luck^39^. Future research could thus aim to investigate how perceptions are affected by how underlying wealth disparities are initially created. Moreover, the potential for the identified fairness ideals to emerge in, and shape, other decision-making contexts involving wealth inequality warrants exploration. Such investigations could advance existing research^40^ on how the strategies impact important societal choices, such as trust, co-operation and coordination.

The distinctive Moral Opportunist motivation additionally requires further investigation. Moral Opportunism is prosocial in that participants who follow this strategy always select between two motivations that reduce inequality between participants. However, people adopting this motivation selectively choose the financially least taxing option. In this way, our results support previous findings demonstrating that the underlying strategy for this approach is in fact self-interest^39^. In the current experiment, participants who followed the Moral Opportunist motivation in the DG not only alternated between Total and Table Egalitarianism, but also largely switched to a Table Egalitarian strategy in the UG. As mentioned previously, we propose that this behavior reflects selfish tendencies influenced by strategic considerations. Future investigation into the neural processes underlying this motivation could offer extra insight, including into the extent to which the decision motivations found here are a possible manifestation of underlying stable traits^32^.

Several limitations of our study are worth discussing. The wealth differences introduced between the participants were relatively small. Nonetheless, despite the subtle disparities, behavior in our study aligns with often observed patterns whereby wealthier individuals give away more than those with less wealth^29,31^. It thus appears that the amount of wealth was not treated as a random factor by our participants, suggesting that even minor wealth differences can have meaningful effects in the examination of distributive preferences under unequal conditions. Similarly, our game stakes simulate small economic choices. Participants’ decisions were, however, consequential and had practical implications for the players. Furthermore, meta-analysis of DG and UG studies has shown that stake sizes have minimal effect on task behavior^41^, lending support to our design.

As evidence suggests that differences in prevailing views of fairness correlate with public policy across countries^42^, our findings provide important lessons on both human behavior as well as policy and law formulation across contexts. A prime example is taxation policies which prominently display the ongoing tension between Total and Table Egalitarian ideals. Total Egalitarians may include those who advocate for progressive tax systems that impose higher rates on wealthier individuals. Conversely, proponents of Table Egalitarianism may endorse flat tax rates where everyone contributes the same percentage of their income in order to achieve an equitable distribution of the tax burden. Debates on the construction of inheritance laws provide another relevant example. Total Egalitarians might advocate for substantial inheritance taxes to diminish intergenerational wealth accumulation and reduce economic disparities. In contrast, Table Egalitarians may prefer laws which do not place such burden on the heirs of wealthy estates. Furthermore, within each of these examples individuals may also lean towards policy support based on a Pro-Self or Moral Opportunist perspective, depending on the contextual factors at play. Our findings thus highlight divergent priorities in the pursuit of fairness and the complex decisions confronting individuals, including those involved in shaping laws and policies, when addressing real-world wealth inequities. The choice between these strategies often hinges on the specific context in which these decisions are made emphasizing the nuanced nature of these debates.

Collectively, our results highlight three key facets of distributive preferences in situations of wealth inequality, namely: the Distributor’s self-interest, their motivation to reduce inequalities unrelated to the immediate distributive choice, and the degree to which the receiving party has influence on the distribution. Importantly, there are substantial individual differences across these three aspects. The significance of this structured understanding becomes evident when considering the need to address increasingly polarized political opinions on wealth inequality and rising global economic disparities^2^. Our analysis thus highlights the highly individualized nature of distributive preferences, revealing that inequality does not elicit uniform fairness concerns across individuals.

## Methods

### Participants

111 participants residing in the United States of America (US) were recruited via Amazon Mechanical Turk (MTurk). Three participants were excluded from analysis because they did not follow instructions correctly. Therefore, data from 108 participants was analyzed (*M*_*age*_ = 35.40 ± 10.76 years, range 19–64, 44 females). The Ethical Committee Social Sciences of the Radboud University, The Netherlands (ECSW2017-2306-552 Huijsmans Sanfey) approved the study protocol with research performed in accordance with the Declaration of Helsinki, and informed consent was obtained from all participants before commencing the experiment.

The experimental procedures were programmed in Neurotask Scripting Beta (www.neurotask.com)^43^. Participants received a flat rate fee of $1 for their participation and took approximately 15 min to complete the task. Additionally, as further detailed below, for each participant the outcome of one random trial was selected and paid as a bonus on top of the flat-fee of $1. On average, participants received a bonus of $1.62 ± $1.16 (range $0.19–$3.80).

### Experimental procedure

In fixed order, participants first played the Dictator Game (DG) and then the Ultimatum Game (UG). Participants always played in the role of Distributor in the DG and Proposer in the UG – for convenience, we will jointly refer to both of these roles as ‘Distributor’ in the respective games henceforth. For each trial as well as across each game, participants played with a different partner, and were informed that their choices would affect both their own and others’ pay-outs. Participants also played in the role of Receiver in a separate session one to two weeks later, however we will only focus on decisions in the Distributor roles in the current paper.

To introduce differing wealth between the players, both Distributors and Receivers were endowed with extra bonus money (which we refer to here as *wealth*) at the start of each game round. Three different wealth levels were chosen to reflect a theoretically meaningful range of inequality, corresponding to a standard GINI coefficient, while ensuring that differences between pre-existing experimental wealth were notable enough to elicit distinct behavioral responses in an online environment. We also sought to examine whether participants behaved consistently across varying degrees of wealth disparity, where achieving equality requires larger sacrifices in conditions of high inequality. Accordingly, Distributors were endowed with either $0.19, $0.75 or $3.00, which we refer to as Distributor Wealth (DW). DW was blocked for each participant, but counterbalanced between participants, ensuring each DW level appeared in every possible position (first, second or third) across different participants. Participants were aware that their wealth would change throughout the game. In each block of DW, participants were paired with nine new Receivers, each of whom also received an endowment of $0.19, $0.75 or $3.00, which we term Receiver Wealth (RW). Distributors played three times each with a Receiver having a RW of $0.19, $0.75, or $3.00. The order of RW was randomized within each DW block. In total, this design resulted in 27 trials in the DG and 27 trials in the UG, with Distributors making decisions across all combinations of the 3 DW levels and 3 RW levels in each game, with different Receivers for every trial.

For each trial, participants saw the relevant DW and RW, and then made allocation decisions about the endowment of $1. They indicated how much they wanted to give the Receiver in increments of $0.10 by moving a bar on a slider using the arrow keys of their keyboard. To enhance credibility, participants were asked to enter their initials which would be presented to the Receiver. During both the DG and UG, Distributors were shown their initials and Receiver was referred to as ‘Other’, as they had not yet played. Participants were aware they would never play with the same partner twice. We instructed participants that their choices would influence the payout of both themselves and the Receiver. Responses of Distributors and Receivers were matched afterwards based on the Receivers’ decisions in the later session, and one choice was randomly selected for payout. For the trial that was selected, both the Distributor and Receiver received the wealth they were allocated on that trial, as well as the outcome of their DG or UG choice. The terms ‘DG’ or ‘UG’ were never mentioned to participants.

After completing the DG and UG tasks, participants completed a demographic questionnaire, providing information about their age, gender (male, female, other) and education (1 = ‘Did not complete high school’, 2 = ‘High school’, 3 = ‘College Graduate’, 4 = ‘Graduate school’). Participants also rated their political orientation on a Likert Scale from 1 to 6 (1 indicating ‘Democrat’ and 6 indicating ‘Republican’), and religiosity on a Likert Scale from 1 to 6 (1 indicating ‘Not religious at all’ and 6 indicating ‘Extremely religious’). We further assessed subjective relative socioeconomic status (SES) by using the McArthur Social Ladder, in which participants are asked to place themselves on a ten-rung ladder representing the social hierarchy of the US^44^. Additionally, to gather insights into real-life economic wealth, participants were asked to estimate their personal annual income, the annual income of their household, and the annual income of their family while they were growing up. Response options were structured as follows: <$15,000, $15,001–$25,000, $25,001–$35,000, $35,001–$50,000, $50,001–$75,000, $75,001–$100,000, > $150,000. Lastly, participants were asked to elaborate on their decision-making strategies in the DG and UG via two open-ended questions, which allowed them to provide qualitative insights into their motivations during the games.

### Analyses

#### Mean giving behavior

To investigate wealth effects on allocations in the context of both DG and UG, we analyzed mean giving behavior using Repeated Measures ANOVAs. As the dependent variable we used the amount given to the Receiver (in dollars ($)) and as within-subjects variables we added Distributor Wealth (DW: $0.19, $0.75, $3.00), Receiver Wealth (RW: $0.19, $0.75, $3.00), and game (DG: Dictator Game, UG: Ultimatum Game). This allowed us to investigate if mean giving behavior was impacted by these different conditions. All analyses were done in R Studio Version ‘2021.9.0.351’ (2021).

#### Computational model

To allow us to investigate individual differences that underlie mean giving behavior in a quantitative way, we utilized a computational modeling approach (elsewhere also termed ‘structural modeling’^45^). More specifically, we developed a utility model that formalized two fairness ideals, Table Egalitarianism and Total Egalitarianism. By adapting previous models of inequality aversion, such as Fehr and Schmidt^18^ and van Baar et al.^34^, our model balances the two fairness ideals against monetary self-interest in the Distributor’s choice as to how to split the $1 endowment (the Table Stake).

The first fairness ideal, Table Egalitarianism, aims to reduce Table Inequality, that is, any inequality arising from the Table Stake. This is achieved by dividing the Table Stake equally, disregarding any pre-existing wealth that exists for either player. This model closely aligns with the inequality aversion proposed by Fehr and Schmidt (1999), and is formalized as follows:

2 $${{\text{I}}_{{\text{Table}}}}{\text{}}={\left( {\frac{{{{\text{m}}^{\text{D}}}}}{{{{\text{m}}^{\text{D}}}+{{\text{m}}^{\text{R}}}}} - 0.5} \right)^2}$$

Here, I_Table_ is the Table Inequality reduced by participant’s behavior. m^D^ represents the amount of money kept by the Distributor, and m^R^ the amount of money given to the Receiver. In our paradigm, as the Distributor always splits $1, m^D^ + m^R^ always equals 1. As mentioned above, Table Egalitarianism is achieved when I_Table_ is 0, meaning m^D^ is 0.5.

The second fairness ideal, Total Egalitarianism, aims to reducing Total Inequality. To achieve this, the aim is to use the Table Stake to maximally equalize inequality, including those arising from any initial wealth differences between both players. The total inequality reduced by participant’s behavior is formalised as:

3 $${{\text{I}}_{{\text{Total}}}}{\text{}}={\left( {\frac{{\left( {{\text{DW}} - {\text{RW}}} \right)+{{\text{m}}^{\text{D}}}~}}{{\sqrt {\left| {{\text{DW}} - ~{\text{RW}}} \right|} ~+{{\text{m}}^{\text{D}}}+{{\text{m}}^{\text{R}}}}} - 0.5} \right)^2}$$

Here, I_Total_ indicates the extent to which the giving behavior of the Distributor affects total inequality between the players, including those induced by the pre-existing wealth levels. DW represents the wealth amount received by the Distributor, and RW refers to the wealth amount allocated to the Receiver. When there is no wealth inequality between the Distributor and Receiver, DW - RW is zero. In these cases, I_Total_ becomes I_Table_, as seen in Eq. (2). Importantly, we assume that as the wealth difference between the DW and RW becomes larger, the weight of the inequality also becomes larger. To account for this, we include a square root in the denominator. This adjustment amplifies the effect of greater disparities, ensuring that greater wealth differences are treated as disproportionately impactful. Additionally, I_Total_ accounts for the relative positions of the Distributor and Receiver, addressing the asymmetry in how wealth disparities influence fairness perceptions from the Distributor’s perspective. By incorporating these elements, I_Total_ captures context-sensitive perceptions of inequality.

Next, in order to examine whether both of these ideals are present in our sample of interest, we built a utility model, termed the ‘Two Norms’ model. This model fits two free parameters that together represent the two aforementioned fairness ideals as well as the preference of simply always allocating all of the amount to oneself (selfish preference). Assuming the participant’s choice is represented by the alternative that yields the highest utility, in our model we formalize utility in situations ranging from equality to inequality as seen in Eq. (1).

In this model, one free parameter (φ) represents selfish tendencies, which weighs monetary self-interest against social preferences. When φ is zero, this indicates minimum selfish preferences, and full preferences to divide equally. For the wealth levels under investigation here, there is no variability in giving behavior for φ values larger than 0.5 (see: Fig. S1A). For φ values larger than 0.5, the model gives maximum utility to keeping the entire Table Stake. Therefore, we bound the φ parameter between 0 and 0.5 (0 < φ < 0.5).

The second free parameter represents the specific social decision strategy (ϑ), reflecting the trade-off between the two fairness rules as applied to the Distributor’s choice. When ϑ is zero, the model follows Total Egalitarian preferences. When ϑ is one, the model follows Table Egalitarian preferences. As detailed above, m^D^ refers to the amount of the $1 endowment that the Distributor chooses to keep for themselves.

We compared the Two Norms model with two other models that each hypothesize that participants’ social preferences are dependent exclusively on only one of the two fairness ideals.

4 $${{\text{U}}_{({\text{Table}})}}\,=\,{\text{}\phi}*{\text{ }}{{\text{m}}^{\text{D}}} - ({\text{1}} - {\text{}\phi}){\text{ }}*{\text{ }}\left( {{{\text{I}}_{{\text{Table}}}}} \right)$$

5 $${{\text{U}}_{({\text{Total}})}}\,=\,{\text{}\phi}*{\text{ }}{{\text{m}}^{\text{D}}} - ({\text{1}} - {\text{}\phi}){\text{ }}*{\text{ }}\left( {{{\text{I}}_{{\text{Total}}}}} \right)$$

For the Table model, this is the same as the Two Norms model, but for the specific situation in which ϑ equals one. Similarly, for the Total model, this is the same as the Two Norms model, but for the unique situation that ϑ equals zero. Comparing these three models allows a test of whether there is significant distribution of both Table and Total Egalitarian preferences across participants, thereby determining whether the Two Norms model is best fit to the data. Importantly, we apply these models to choices made in both DG and UG, helping provide a controlled framework for comparing behavior across the two decision contexts.

#### Model fitting

To fit the three models to the data, we employed a series of nested loops, iterating over each individual Distributor choice for the DG and UG separately, and varying the free parameters (φ and ϑ) of each model. Specifically, the three models were categorised by different ϑ values, such that when ϑ equals zero, we applied the Total Egalitarian model, when ϑ equals one, we applied the Table Egalitarian model and when ϑ equals neither zero or one, we utilised the Two Norms model. For each game type and theta value, we used the *least*_*squares* routine in *Scipy* to minimize the sum of squared error between the model’s behavioral prediction and the actual behavior of the participants over the 27 trials collected per game type. For each participant, to avoid finding a local minimum, the model fitting procedure was initialized at 10 000 random points in the ϑ and φ parameter space within the bounds (0 ≤ φ ≤ 0.5 and 0 ≤ ϑ ≤ 1.0). We selected the parameters of ϑ and φ that resulted in the least sum of squared errors for each participant per game type. When two or more iterations of this procedure resulted in equally good fits, the first occurring iteration was selected.

### Model comparison

To assess which model best explained participants’ decision behavior, we measured and compared the three models’ respective performance using both the Bayesian Information Criterion (BIC)^46^ as well as Akaike Information Criterion (AIC)^47^. Both measures provide complementary information about the fit of different models to the data, with the ‘best’ model being the one with the lowest AIC and/or BIC value. In order to compare whether the ‘Two Norms’ model is the best overall among the three models, we computed AIC and BIC values per participants and performed Wilcoxon signed-rank tests on subject-wise AIC and BIC differences separately, for both the DG and UG. AIC and BIC are respectively defined as:

6 $${\text{AIC}}\,=\,n.{\text{ ln }}\left( {{\text{SSE}}/n} \right)\,+\,k.{\text{ 2}}$$

7 $${\text{BIC}}\,=\,n{\text{ }}.{\text{ln}}\left( {{\text{SSE}}/n} \right)\,+\,k{\text{ }}.{\text{ln}}(n)$$

where *SSE* represents the residual sum of squares (i.e. the sum over squared differences between model prediction and actual behavior), *n* represents the number of observations (trials) and *k* represents the number of free parameters in the model (ϑ and/or φ).

Notably, 16 participants exhibited consistent behavior across the DG, UG and/or the entire experiment. Specifically, 16 participants in the DG and 2 participants in the UG always chose to keep the $1 endowment. For these participants, their behavior resulted in an undefined logarithm of zero. Accordingly, to prevent excluding any participants from model comparison, the computation of AIC and BIC included a small constant equal to 0.000001 for all participants. This ensured that all participants were included in model fitting procedures as well as AIC and BIC comparisons without significantly impacting participants whose behavior were not perfectly explained by any of the models.

### Model performance

To ensure our model was robustly identifiable, we conducted parameter recovery analyses for the DG and UG (*SI Materials and Methods*). In both analyses, the relationship between true and recovered φ was high (DG: *r* = 0.99; *p* < 0.001; UG: *r* = 0.99; *p* < 0.001). Similarly, the relationship between true and recovered ϑ was strong (DG: *r* = 0.87; *p* < 0.001; UG: *r* = 0.86; *p* < 0.001) (see: Fig. S1B and C).

### Clustering participants by decision strategy

To better visualize and understand the potential motivations underlying decisions in the DG and UG, we clustered our participants in a decision strategy space. To do this, we examined ϑ and φ in relation to each other in a 2D parameter space allowing us to form unbiased and objective clusters that represent different principled decision strategies. To this end, we applied the model-driven hierarchical clustering approach used in van Baar et al.^34^ and then grouped participants as to how they fell within cluster boundaries.

Specifically, we simulated choice data for ϑ values ranging from zero to one in steps of one hundred, and formed pairs with φ values ranging from zero to 0.5 in equally sized steps, resulting in a total of 10,201 (101 × 101) unique pairs of ϑ and φ values. For each unique combination of parameter values, we simulated choice data for each unique interaction pair (DW × RW, nine pairs). Next, we computed the pairwise squared Euclidian distance for each combination, and used hierarchical clustering from the *Scipy* package in Python to obtain four parsimonious clusters. Therefore, the word ‘cluster’ in this paper is used to refer to a specific area in the parameter space. Qualitatively, the clusters align with theoretical predictions of the four motivations we aimed to capture.

Then, we calculated for each participant, for the DG and UG seperately, to which cluster their behavior most closely resembled, based on their estimated ϑ and φ parameters. We refer to this as their ‘decision strategy’. Therefore, within participants, strategy can differ between DG and UG. One advantage of this method is illustrative, in that it allows us to reduce the complexity of the 2D parameter space and to think about decision strategies in a discrete way. Another advantage of this method is that it enables us to do analyses that allow for interpretations about movement between strategy groups and through the 2D space. However, we do not mean to imply that the transition in strategy across the boundaries of cluster is discrete. We propose that by moving through the parameter space, the decision strategy of participants may gradually change.

### Decision strategies across contexts

In order to obtain a comprehensive understanding of the strategies used in the DG and UG, we investigated the frequencies of decision strategies in DG as compared to UG, using Pearson’s chi-squared tests. This allowed us to examine our hypothesis that the strategic considerations underlying giving behavior in the UG (Camerer, 2011), would affect strategy choice in each game. Specifically, we hypothesized that the Pro-Self strategy would occur more frequently in the DG compared to the UG, and that Table Egalitarianism would be a dominant strategy in the UG.

Next, we sought to examine the strength and direction of the relationship underlying the decision strategies (i.e. φ and ϑ values) by calculating Pearson’s product correlations. In so doing, we were able to test our hypothesis that changing behavior between the DG and UG was motivated by changes in strategic considerations.

As confirmatory analysis of the four strategies, we conducted Welch’s ANOVA to examine behavior for each cluster in the DG and UG separately. We anticipated distinct average giving behavior per strategy, and specifically expected average giving behavior to be approximately $0.50 for Total and Table Egalitarianism, and roughly $0.00 for Pro-Self.

Finally, we investigated the association between switching strategies between games, with reference to the initial strategy used (in the DG). To do this, we conducted a Pearson’s chi-square test to assess consistency of decision strategies. This allowed us to thus test our prediction that when individuals exhibit prosocial behavior in the DG, their preferences towards prosociality remains stable across both games. In this regard, we hypothesized a correlation between ϑ values in the DG and ϑ values in the UG. We also expected that participants classified as either Total or Table Egalitarians in the DG would continue to act in alignment with the same motivation in the UG. Conversely, we anticipated that a Pro-Self strategy in the DG would be associated with a shift to a different motivation in the UG.

### Demographic variables

Lastly, we conducted exploratory analyses to investigate the relationship between the parameters in our experiment and socio-demographic characteristics of participants. As independent variables, we included age, gender, education, subjective relative SES, political beliefs, religiosity, and self-reported personal, household and family income.

## Electronic Supplementary Material

Below is the link to the electronic supplementary material.

Supplementary Material 1

## Data Availability

The datasets generated during and analysed during the current study are available at the following OSF repository: https://osf.io/f5zna/.
