# Supplementary material for: Competing fairness ideals underlie wealth inequality across decision contexts
Source: Sci Rep. 2024 Dec 30;14:31882. doi: 10.1038/s41598-024-83361-z (PMC11685827; doi:10.1038/s41598-024-83361-z)
Supplement: Supplementary file 1 — Supplementary Material 1 [file 41598_2024_83361_MOESM1_ESM.docx]

***Supplementary Materials And Methods***

***for***

**Competing Fairness Ideals Underlie Wealth Inequality**

**Across Decision Contexts**

Inge Huijsmans,^1a^ Sarah Vahed,^1a^* Cătălina E. Rățală,^1^ Alberto Llera,^1,2^ Alan G. Sanfey^1,3^

^1^ Donders Institute for Brain, Cognition and Behavior, Radboud University, Nijmegen, The Netherlands

^2^ Department of Cognitive Neuroscience, Radboud University Nijmegen Medical Centre, Nijmegen, Netherlands

^3^ Behavioural Science Institute, Radboud University, Nijmegen, The Netherlands

^a^ Joint first author. These authors contributed equally to this work.

* Corresponding author: sarah.vahed@donders.ru.nl

*Experimental Instructions*

*
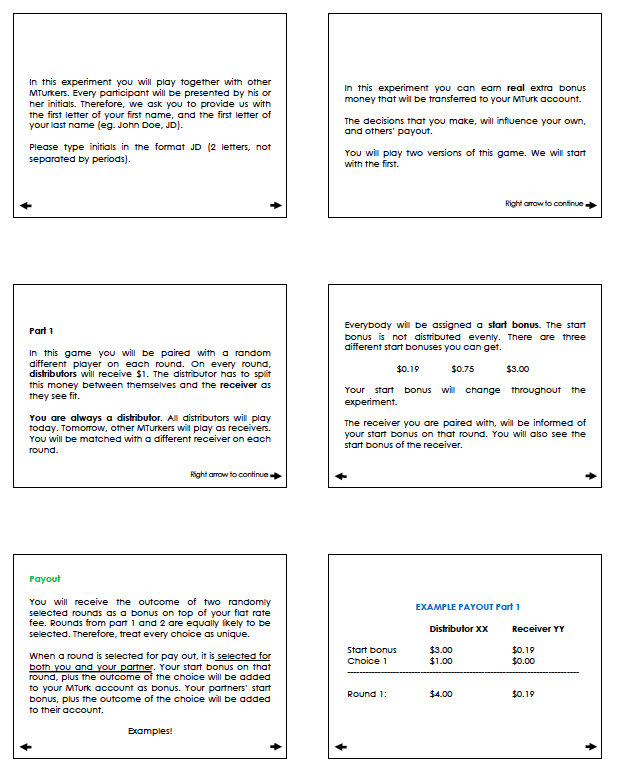
*

*
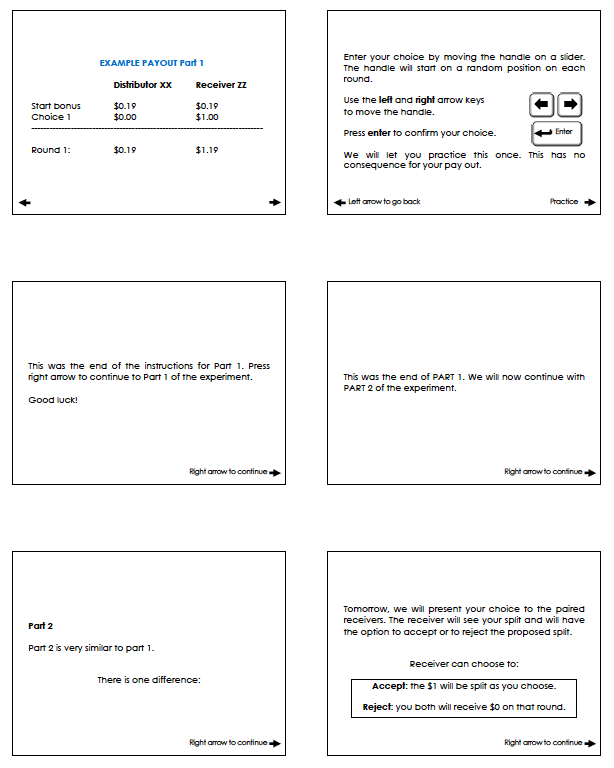
*

*
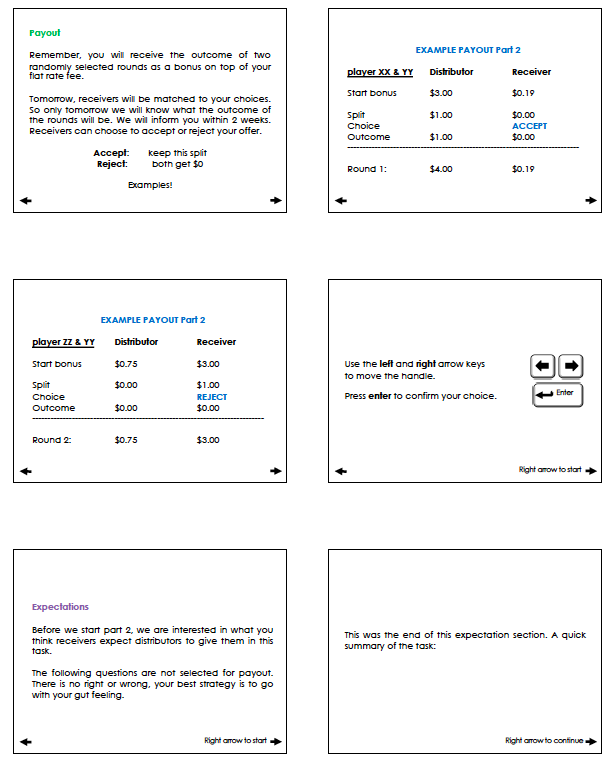
*

*
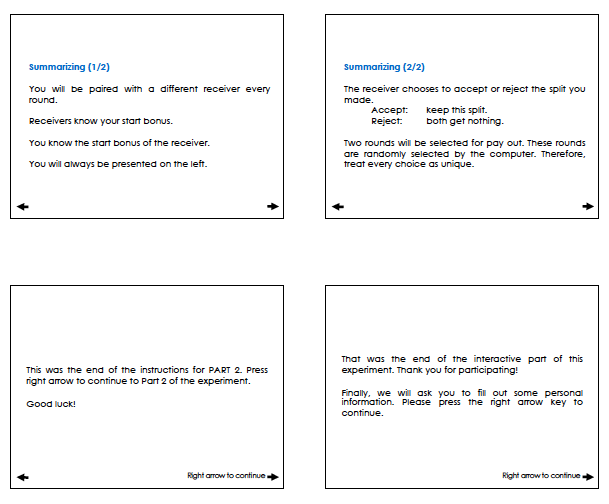
*

*Supplementary SI Methods and Materials*

*Parameter Recovery Process*

We calculated a Pearson’s correlation coefficient to assess the relationship between true and recovered parameters. First, we simulated choice data from 108 imaginary participants using random ϑ and φ parameter values across the levels of DW and RW, resulting in a set of nine DW-RW combinations. For each participant, we executed three trials per condition, resulting in 27 simulated choices. Next, we estimated ϑ and φ parameters for the simulated participants by running 1 000 iterations of the model fitting procedure described above. The process was run twice, once for the DG and once for the UG.

In both analyses, the relationship between true and recovered φ was very high (DG: *r* = 0.99; *p* < .001; UG: *r* = 0.99; *p* < .001). Similarly, the relationship between true and recovered ϑ was strong (DG: *r* = 0.87; *p* < .001; UG: *r* = 0.86; *p* < .001). Further inspection of the recovered ϑ values revealed that for true φ values between 0 and 0.4 the correlation between true and recovered ϑ is near perfect (DG: *r* = 0.98; *p* < .001; UG: *r* = 0.98; *p* < .001). For φ values between 0.4 and 0.5, the relationship between true and recovered ϑ exhibited variation (DG: *r* = 0.54, *p* = 0.03; UG: *r* = 0.31, *p* = 0.22). This suggests that participants with high monetary self-interest prioritize social preference less in their decisions. Lastly, we simulated choice data with the recovered ϑ and φ parameters, and the correlations between simulated choice datasets from the recovered and the true parameters is *r_DG_* = 1.00 (p <.001) and *r_UG_* = 1.00 (p <.001). For parameter values reported in this paper, we include the full range of ϑ (i.e. 0 – 1.0) and φ (i.e. 0 - 0.5). *Figure S1B* and *C* illustrate the relationship between the true and recovered parameters for DG and UG respectively.

*Supplementary Figure S1. Model Simulation and Parameter Recovery.*


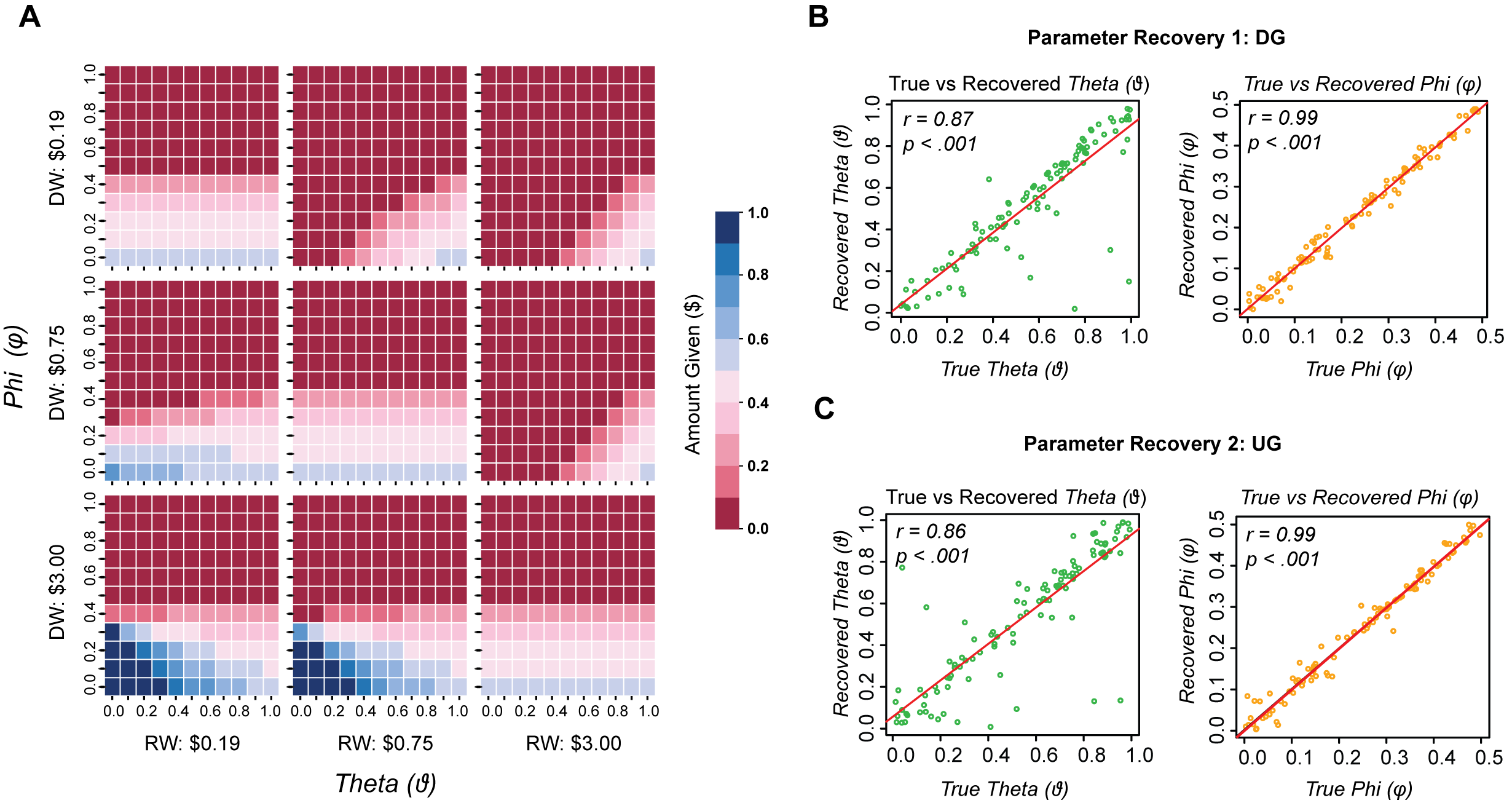


*(A) Simulation of choices based on φ (phi) and ϑ (theta) parameters in the Two Norms Model across DW-RW combinations, assuming that individuals make decisions to maximize their utility. For φ values larger than 0.5, there is no variability in giving behaviour, and the model assigns maximum utility to keeping the entire Table Stake. Parameter Recovery Analyses for Dictator Game (B) and Ultimatum Game (C) reflecting true and recovered φ (phi) and ϑ (theta) parameters, with ϑ ranging from 0.0 to 1.0 and φ from 0.0 to 0.5, respectively.*

*Table S1: Pairwise Interactions between each DW-RW pair in the DG*

| **Contrast** |  |  | |  | **Estimate** | **SE** | **df** | **t-ratio** | **p-value** |
| --- | --- | --- | --- | --- | --- | --- | --- | --- | --- |
| **DW** | **RW** |  | **DW** | **RW** |  |  |  |  |  |
| 0.19 | 0.19 | - | 0.75 | 0.19 | -0,04599 | 0,0162 | 107 | -2,833 | 0,1181 |
| 0.19 | 0.19 | - | 3 | 0.19 | -0,12778 | 0,027 | 107 | -4,735 | 0,0002 |
| 0.19 | 0.19 | - | 0.19 | 0.75 | 0,13673 | 0,0157 | 107 | 8,695 | <.0001 |
| 0.19 | 0.19 | - | 0.75 | 0.75 | 0,01759 | 0,0133 | 107 | 1,319 | 0,9234 |
| 0.19 | 0.19 | - | 3 | 0.75 | -0,12315 | 0,0266 | 107 | -4,626 | 0,0003 |
| 0.19 | 0.19 | - | 0.19 | 3 | 0,22963 | 0,0223 | 107 | 10,299 | <.0001 |
| 0.19 | 0.19 | - | 0.75 | 3 | 0,22284 | 0,0219 | 107 | 10,186 | <.0001 |
| 0.19 | 0.19 | - | 3 | 3 | 0,02407 | 0,0173 | 107 | 1,391 | 0,8989 |
| 0.75 | 0.19 | - | 3 | 0.19 | -0,08179 | 0,0236 | 107 | -3,464 | 0,021 |
| 0.75 | 0.19 | - | 0.19 | 0.75 | 0,18272 | 0,0235 | 107 | 7,778 | <.0001 |
| 0.75 | 0.19 | - | 0.75 | 0.75 | 0,06358 | 0,0128 | 107 | 4,986 | 0,0001 |
| 0.75 | 0.19 | - | 3 | 0.75 | -0,07716 | 0,0224 | 107 | -3,439 | 0,0227 |
| 0.75 | 0.19 | - | 0.19 | 3 | 0,27562 | 0,0305 | 107 | 9,039 | <.0001 |
| 0.75 | 0.19 | - | 0.75 | 3 | 0,26883 | 0,0297 | 107 | 9,047 | <.0001 |
| 0.75 | 0.19 | - | 3 | 3 | 0,07006 | 0,0197 | 107 | 3,559 | 0,0156 |
| 3 | 0.19 | - | 0.19 | 0.75 | 0,26451 | 0,0322 | 107 | 8,226 | <.0001 |
| 3 | 0.19 | - | 0.75 | 0.75 | 0,14537 | 0,0255 | 107 | 5,691 | <.0001 |
| 3 | 0.19 | - | 3 | 0.75 | 0,00463 | 0,0125 | 107 | 0,37 | 1 |
| 3 | 0.19 | - | 0.19 | 3 | 0,35741 | 0,037 | 107 | 9,656 | <.0001 |
| 3 | 0.19 | - | 0.75 | 3 | 0,35062 | 0,0371 | 107 | 9,451 | <.0001 |
| 3 | 0.19 | - | 3 | 3 | 0,15185 | 0,0222 | 107 | 6,854 | <.0001 |
| 0.19 | 0.75 | - | 0.75 | 0.75 | -0,11914 | 0,017 | 107 | -7,028 | <.0001 |
| 0.19 | 0.75 | - | 3 | 0.75 | -0,25988 | 0,0322 | 107 | -8,061 | <.0001 |
| 0.19 | 0.75 | - | 0.19 | 3 | 0,0929 | 0,0128 | 107 | 7,275 | <.0001 |
| 0.19 | 0.75 | - | 0.75 | 3 | 0,08611 | 0,0149 | 107 | 5,78 | <.0001 |
| 0.19 | 0.75 | - | 3 | 3 | -0,11265 | 0,0187 | 107 | -6,016 | <.0001 |
| 0.75 | 0.75 | - | 3 | 0.75 | -0,14074 | 0,0254 | 107 | -5,541 | <.0001 |
| 0.75 | 0.75 | - | 0.19 | 3 | 0,21204 | 0,0242 | 107 | 8,766 | <.0001 |
| 0.75 | 0.75 | - | 0.75 | 3 | 0,20525 | 0,0236 | 107 | 8,703 | <.0001 |
| 0.75 | 0.75 | - | 3 | 3 | 0,00648 | 0,0158 | 107 | 0,41 | 1 |
| 3 | 0.75 | - | 0.19 | 3 | 0,35278 | 0,0378 | 107 | 9,331 | <.0001 |
| 3 | 0.75 | - | 0.75 | 3 | 0,34599 | 0,0377 | 107 | 9,182 | <.0001 |
| 3 | 0.75 | - | 3 | 3 | 0,14722 | 0,0219 | 107 | 6,737 | <.0001 |
| 0.19 | 3 | - | 0.75 | 3 | -0,00679 | 0,0093 | 107 | -0,73 | 0,9982 |
| 0.19 | 3 | - | 3 | 3 | -0,20556 | 0,0237 | 107 | -8,68 | <.0001 |
| 0.75 | 3 | - | 3 | 3 | -0,19877 | 0,0234 | 107 | -8,504 | <.0001 |
| Note: *P value adjustment: Tukey method for comparing a family of 9 estimates* | | | | | | | |  |  |

*Table S2: Pairwise Interactions between each DW-RW pair in the UG*

| **Contrast** |  |  | |  | **Estimate** | **SE** | **df** | **t-ratio** | **p-value** |
| --- | --- | --- | --- | --- | --- | --- | --- | --- | --- |
| **DW** | **RW** |  | **DW** | **RW** |  |  |  |  |  |
| 0.19 | 0.19 | - | 0.75 | 0.19 | -0,08673 | 0,0151 | 107 | -5,745 | <.0001 |
| 0.19 | 0.19 | - | 3 | 0.19 | -0,21019 | 0,0226 | 107 | -9,301 | <.0001 |
| 0.19 | 0.19 | - | 0.19 | 0.75 | 0,14753 | 0,01191 | 107 | 12,382 | <.0001 |
| 0.19 | 0.19 | - | 0.75 | 0.75 | 0,01204 | 0,00839 | 107 | 1,434 | 0,8821 |
| 0.19 | 0.19 | - | 3 | 0.75 | -0,18025 | 0,02158 | 107 | -8,354 | <.0001 |
| 0.19 | 0.19 | - | 0.19 | 3 | 0,25864 | 0,01878 | 107 | 13,769 | <.0001 |
| 0.19 | 0.19 | - | 0.75 | 3 | 0,22963 | 0,019 | 107 | 12,086 | <.0001 |
| 0.19 | 0.19 | - | 3 | 3 | 0,00525 | 0,00836 | 107 | 0,628 | 0,9994 |
| 0.75 | 0.19 | - | 3 | 0.19 | -0,12346 | 0,01559 | 107 | -7,921 | <.0001 |
| 0.75 | 0.19 | - | 0.19 | 0.75 | 0,23426 | 0,02121 | 107 | 11,042 | <.0001 |
| 0.75 | 0.19 | - | 0.75 | 0.75 | 0,09877 | 0,01169 | 107 | 8,447 | <.0001 |
| 0.75 | 0.19 | - | 3 | 0.75 | -0,09352 | 0,01532 | 107 | -6,104 | <.0001 |
| 0.75 | 0.19 | - | 0.19 | 3 | 0,34537 | 0,02859 | 107 | 12,079 | <.0001 |
| 0.75 | 0.19 | - | 0.75 | 3 | 0,31636 | 0,02824 | 107 | 11,203 | <.0001 |
| 0.75 | 0.19 | - | 3 | 3 | 0,09198 | 0,01473 | 107 | 6,243 | <.0001 |
| 3 | 0.19 | - | 0.19 | 0.75 | 0,35772 | 0,02923 | 107 | 12,24 | <.0001 |
| 3 | 0.19 | - | 0.75 | 0.75 | 0,22222 | 0,02146 | 107 | 10,357 | <.0001 |
| 3 | 0.19 | - | 3 | 0.75 | 0,02994 | 0,00946 | 107 | 3,166 | 0,05 |
| 3 | 0.19 | - | 0.19 | 3 | 0,46883 | 0,03657 | 107 | 12,818 | <.0001 |
| 3 | 0.19 | - | 0.75 | 3 | 0,43981 | 0,03562 | 107 | 12,348 | <.0001 |
| 3 | 0.19 | - | 3 | 3 | 0,21543 | 0,02086 | 107 | 10,326 | <.0001 |
| 0.19 | 0.75 | - | 0.75 | 0.75 | -0,13549 | 0,01349 | 107 | -10,044 | <.0001 |
| 0.19 | 0.75 | - | 3 | 0.75 | -0,32778 | 0,02866 | 107 | -11,437 | <.0001 |
| 0.19 | 0.75 | - | 0.19 | 3 | 0,11111 | 0,01249 | 107 | 8,897 | <.0001 |
| 0.19 | 0.75 | - | 0.75 | 3 | 0,0821 | 0,01322 | 107 | 6,208 | <.0001 |
| 0.19 | 0.75 | - | 3 | 3 | -0,14228 | 0,01361 | 107 | -10,458 | <.0001 |
| 0.75 | 0.75 | - | 3 | 0.75 | -0,19228 | 0,02056 | 107 | -9,354 | <.0001 |
| 0.75 | 0.75 | - | 0.19 | 3 | 0,2466 | 0,02067 | 107 | 11,93 | <.0001 |
| 0.75 | 0.75 | - | 0.75 | 3 | 0,21759 | 0,02062 | 107 | 10,554 | <.0001 |
| 0.75 | 0.75 | - | 3 | 3 | -0,00679 | 0,00956 | 107 | -0,71 | 0,9986 |
| 3 | 0.75 | - | 0.19 | 3 | 0,43889 | 0,03586 | 107 | 12,24 | <.0001 |
| 3 | 0.75 | - | 0.75 | 3 | 0,40988 | 0,03554 | 107 | 11,533 | <.0001 |
| 3 | 0.75 | - | 3 | 3 | 0,18549 | 0,02045 | 107 | 9,072 | <.0001 |
| 0.19 | 3 | - | 0.75 | 3 | -0,02901 | 0,01149 | 107 | -2,524 | 0,2326 |
| 0.19 | 3 | - | 3 | 3 | -0,2534 | 0,01959 | 107 | -12,934 | <.0001 |
| 0.75 | 3 | - | 3 | 3 | -0,19877 | 0,0234 | 107 | -8,504 | <.0001 |
| Note: *P value adjustment: Tukey method for comparing a family of 9 estimates* | | | | | | | |  |  |

*Table S3: Model Parameters and related values for Two Norms, Table and Total models for Dictator and Ultimatum Game respectively*

| **Game** | **Model** | **Parameter** | **Constraint** | **Mean** | **M_AIC_** | **M_BIC_** |
| --- | --- | --- | --- | --- | --- | --- |
| **DG** | ***Two Norms*** |  |  |  | -238.55 | -235.96 |
|  |  | Phi | 0 < φ < 0.5 | 0.21 |  |  |
|  |  | Theta | 0 < ϑ < 1.0 | 0.53 |  |  |
|  | Table |  |  |  | -186.05 | -184.75 |
|  |  | Phi | 0 < φ < 0.5 | 0.25 |  |  |
|  |  | Theta | ϑ = 1 | 1 |  |  |
|  | Total |  |  |  | -179.16 | -177.86 |
|  |  | Phi | 0 <φ< 0.5 | 0.30 |  |  |
|  |  | Theta | ϑ = 0 | 0 |  |  |
|  |  |  |  |  |  |  |
| **UG** | ***Two Norms*** |  |  |  | -244.47 | -241.90 |
|  |  | Phi | 0 < φ < 0.5 | 0.07 |  |  |
|  |  | Theta | 0 < ϑ < 1.0 | 0.60 |  |  |
|  | Table |  |  |  | -176.19 | -174.90 |
|  |  | Phi | 0 <φ< 0.5 | 0.11 |  |  |
|  |  | Theta | ϑ = 1 | 1 |  |  |
|  | Total |  |  |  | -123.87 | -122.57 |
|  |  | Phi | 0 <φ< 0.5 | 0.19 |  |  |
|  |  | Theta | ϑ = 0 | 0 |  |  |

*Note.* DG = Dictator Game; UG = Ultimatum Game; AIC = Akaike Information Criterion; BIC = Bayesian Information Criterion.

*Supplementary Figure S2. AIC and BIC Values.*

*
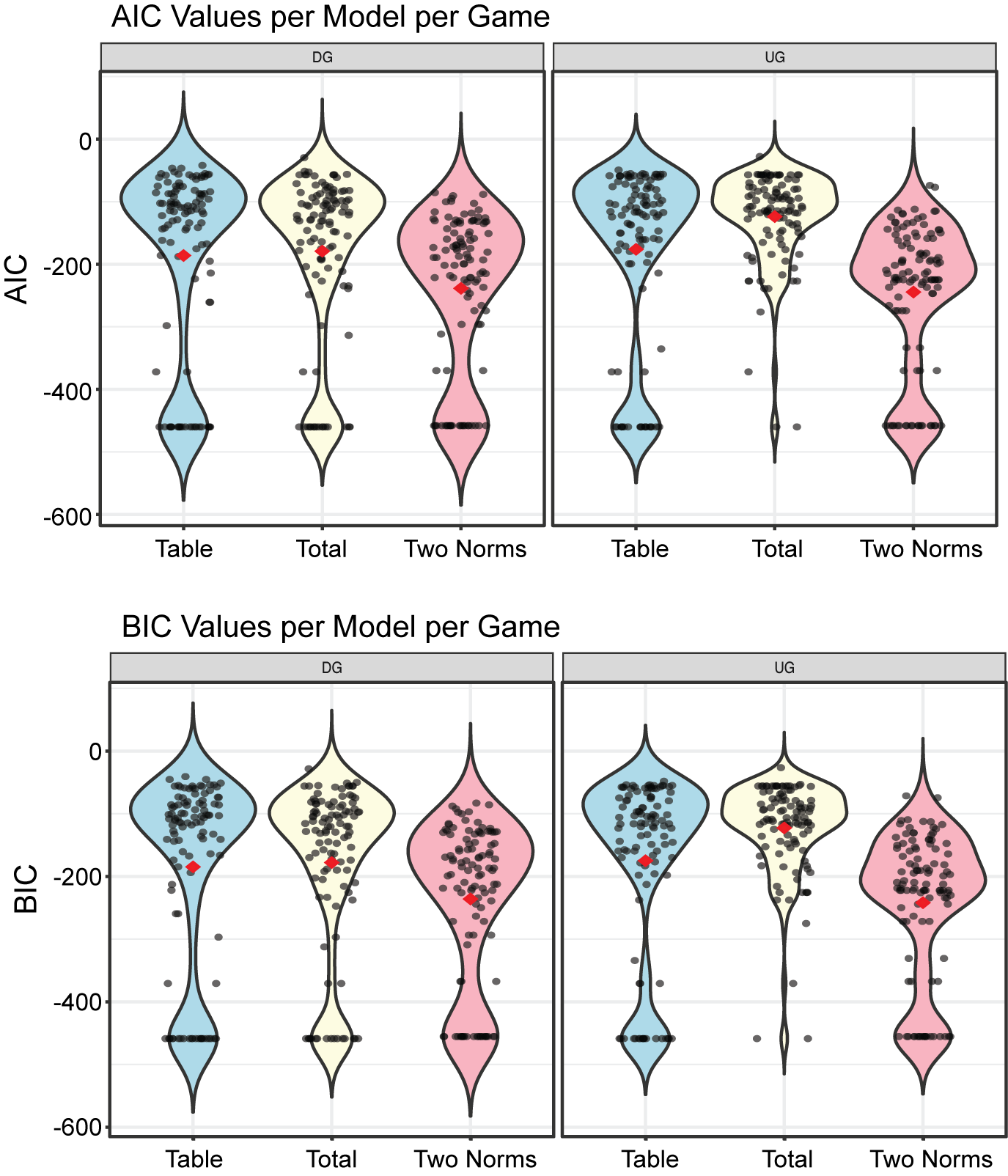
*

*Violin plots depicting AIC (top) and BIC (bottom) values per model: Table; Total and Two Norms. Each dot represents one participant, Red diamond represents mean amount per model.*

*Supplementary Figure S3. Mean Giving Behaviour across Clusters.*

*
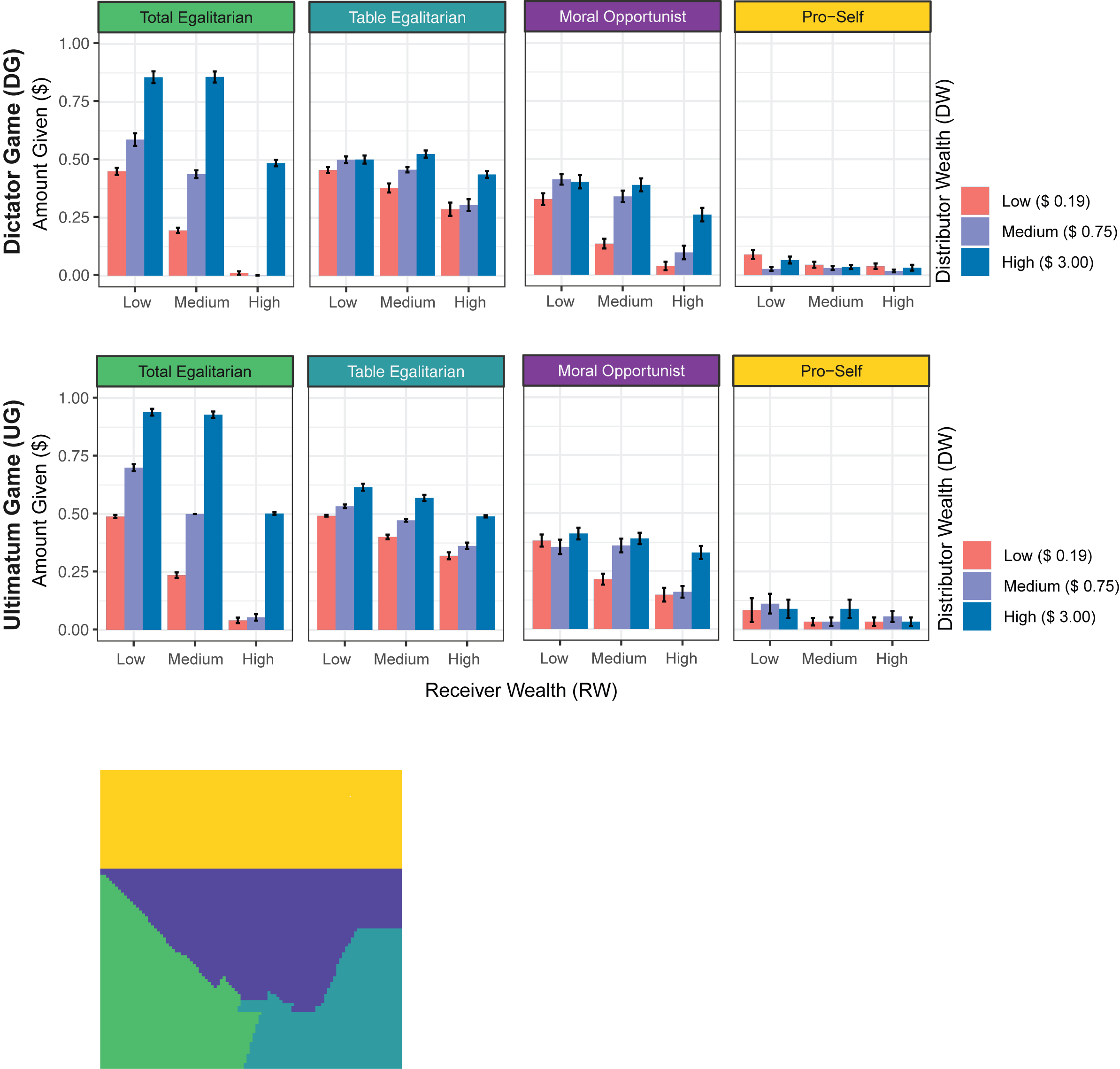
Mean Amount Given ($) for each Receiver Wealth (RW) (x-axis) and Distributor Wealth (DW) condition, per cluster group, in Dictator Game (top) and Ultimatum Game (bottom). For both DW and RW, "Low" = $0.19, "Medium" = $0.75, and "High" = $3.00. Error bars reflect the standard error of the mean.*

*Supplementary Figure S4. Correlation between Parameters and Socio-Demographics.*

*
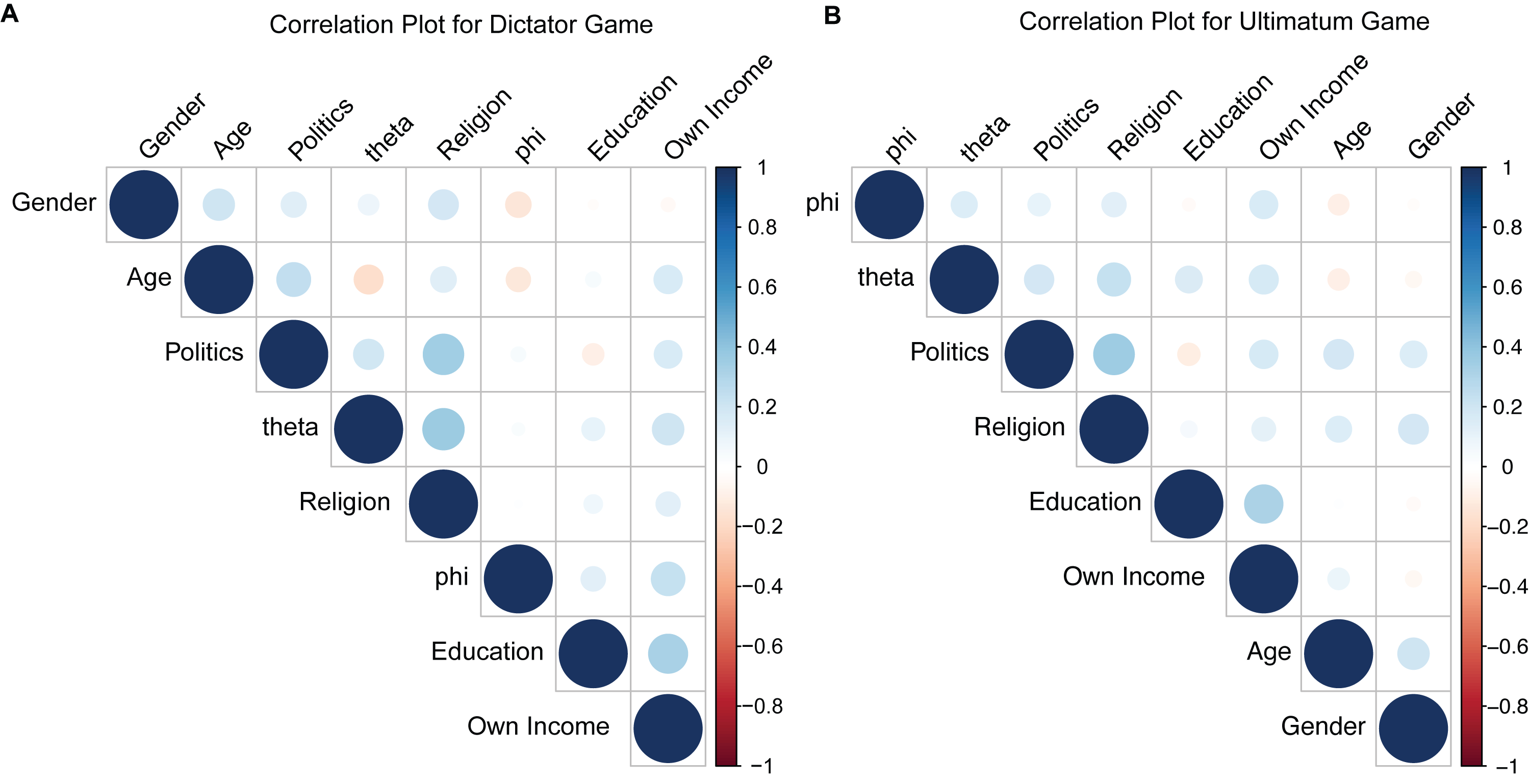
*

*Correlation plots depicting relationships among key variables in the (A) Dictator Game (DG) and (B) Ultimatum Game (UG). The Spearman rank correlation matrix includes model variables (phi and theta) and demographic variables of participants (age, education, gender, income, politics and religion).*

*Table S4: Descriptive statistics*

***DG***

|  |  | **All** | **PS** | **MO** | **TaE** | **ToT** |
| --- | --- | --- | --- | --- | --- | --- |
|  | **count** | 108 | 31 | 20 | 28 | 29 |
| **Gender** | **F**  **M** | 44  64 | 9  22 | 8  12 | 16  12 | 11  18 |
| **Age** | **Mean** | 35.398 | 34.710 | 32.100 | 34.107 | 39.655 |
|  | **s.d.** | 10.765 | 11.281 | 7.133 | 10.331 | 11.404 |
|  |  |  |  |  |  |  |
| **Theta** | **Mean** | 0.532 | 0.514 | 0.584 | 0.778 | 0.280 |
|  | **s.d.** | 0.298 | 0.323 | 0.209 | 0.187 | 0.050 |
| **Phi** | **Mean** | 0.210 | 0.454 | 0.235 | 0.078 | 0.057 |
|  | **s.d.** | 0.177 | 0.047 | 0.036 | 0.179 | 0.081 |
|  |  |  |  |  |  |  |

***UG***

|  |  | **All** | **PS** | **MO** | **TaE** | **ToT** |
| --- | --- | --- | --- | --- | --- | --- |
|  | **count** | 108 | 6 | 11 | 57 | 34 |
| **Gender** | **F**  **M** | 44  64 | 2  4 | 5  6 | 21  36 | 16  18 |
| **Age** | **Mean** | 35.398 | 32.000 | 37.727 | 34.404 | 10.591 |
|  | **s.d.** | 10.765 | 6.344 | 13.514 | 36.912 | 10.319 |
|  |  |  |  |  |  |  |
| **Theta** | **Mean** | 0.600 | 0.575 | 0.737 | 0.777 | 0.264 |
|  | **s.d.** | 0.285 | 0.225 | 0.128 | 0.168 | 0.157 |
| **Phi** | **Mean** | 0.074 | 0.443 | 0.219 | 0.042 | 0.016 |
|  | **s.d.** | 0.113 | 0.062 | 0.058 | 0.037 | 0.025 |
|  |  |  |  |  |  |  |

*Descriptive statistics of individual difference measures, as well as modelling results, in the total sample (‘All’) and split by the strategy groups (PS = Pro-Self; MO = Moral Opportunist; TaE = Table Egalitarian; ToT = Total Egalitarian).*
